# ZNF507 affects TGF-β signaling via TGFBR1 and MAP3K8 activation in the progression of prostate cancer to an aggressive state

**DOI:** 10.1186/s13046-021-02094-3

**Published:** 2021-09-18

**Authors:** Wookbong Kwon, Seong-Kyoon Choi, Daehwan Kim, Hyeon-Gyeom Kim, Jin-Kyu Park, Jee Eun Han, Gil-Jae Cho, Sungho Yun, Wookyung Yu, Se-Hyeon Han, Yun-Sok Ha, Jun Nyung Lee, Tae Gyun Kwon, Dong-Hyung Cho, Jun-Koo Yi, Myoung Ok Kim, Zae Young Ryoo, Song Park

**Affiliations:** 1 Core Protein Resources Center, DGIST, Daegu, Republic of Korea; 2 Division of Biotechnology, DGIST, Daegu, Republic of Korea; 3 School of Life Science, BK21 FOUR KNU Creative Bioresearch, Kyungpook National University, Daegu, Korea; 4 College of Veterinary Medicine, Kyungpook National University, 41566 Daegu, Korea; 5 Department of Brain and Cognitive Sciences, DGIST, Daegu, Republic of Korea; 6 School of Media Communication, Hanyang University, Wangsimni-ro 222, Seongdong- gu, Seoul, South Korea; 7 Department of News-team, SBS (Seoul Broadcasting System), Mokdongseo-ro 161, Yangcheon-gu, Seoul, South Korea; 8 Department of Urology, School of Medicine, Kyungpook National University, Daegu, Korea; 9 Brain Science and Engineering Institute, Kyungpook National University, 41566 Daegu, Republic of Korea; 10 Gyeongsangbuk-do Livestock Research institute, Yeongju, South Korea; 11 Department of Animal Science and Biotechnology, ITRD, Kyungpook National University, 37224 Sangju, Republic of Korea

**Keywords:** Prostate cancer, Metastasis, mCRPC, ZNF507, TGF-β signal

## Abstract

**Background:**

The progression of prostate cancer (PC) to the highly aggressive metastatic castration-resistant prostate cancer (mCRPC) or neuroendocrine prostate cancer (NEPC) is a fatal condition and the underlying molecular mechanisms are poorly understood. Here, we identified the novel transcriptional factor *ZNF507* as a key mediator in the progression of PC to an aggressive state.

**Methods:**

We analyzed ZNF507 expression in the data from various human PC database and high-grade PC patient samples. By establishment of ZNF507 knockdown and overexpression human PC cell lines, we assessed *in vitro* PC phenotype changes including cell proliferation, survival, migration and invasion. By performing microarray with ZNF507 knockdown PC cells, we profiled the gene clusters affected by ZNF507 knockdown. Moreover, ZNF507 regulated key signal was evaluated by dual-luciferase reporter and chromatin immunoprecipitation (ChIP) assays. Finally, we performed xenograft and *in vivo* metastasis assay to confirm the effect of ZNF507 knockdown in PC cells.

**Results:**

We found that *ZNF507* expression was increased, particularly in the highly graded PC. *ZNF507* was also found to be associated with metastatic PC of a high grade. Loss- or gain-of-function–based analysis revealed that *ZNF507* promotes the growth, survival, proliferation, and metastatic properties of PC (e.g., epithelial-mesenchymal transition) by upregulating TGF-β signaling. Profiling of gene clusters affected by *ZNF507* knockdown revealed that *ZNF507* positively regulated the transcription of *TGFBR1*, *MAP3K8*, and *FURIN*, which in turn promoted the progression of PC to highly metastatic and aggressive state.

**Conclusions:**

Our findings suggest that *ZNF507* is a novel key regulator of TGF-β signaling in the progression of malignant PC and could be a promising target for studying the development of advanced metastatic PCs.

**Supplementary Information:**

The online version contains supplementary material available at 10.1186/s13046-021-02094-3.

## Background

The incidence of prostate cancer (PC) has become common and it remains the first men’s cause of cancer-related death in 56 countries [1, 2]. Androgen deprivation therapy (ADT) is widely used as a primary systemic therapy for primary stage PC, however, castration resistance ensues, which eventually leads to an androgen-independent metastatic castration-resistant PC (mCRPC) resulting in brain and bone metastasis [3–9]. These androgen-independent metastatic features of mCRPC can lead to the occurrence of neuroendocrine prostate carcinoma (NEPC), a highly aggressive form, which makes therapeutic options extremely limited [10–12]. Therefore, novel markers that efficiently predict disease progression are urgently needed for PC.

Zinc finger protein 507 (ZNF507) is an ancient, highly conserved C2H2-zinc finger protein, which is thought to regulate transcription [13]. Various studies have implicated ZNF507 as a risk factor for neurodevelopmental disorders and the early development [14–17]. Studies of genetic variations in cancer have indicated that ZNF507 may be relevant in several cancers [18–20]. However, the precise role of ZNF507 in the cancer remains unclear.

Transforming growth factor-β (TGF-β) signaling, which includes canonical and non-canonical pathways, has been linked with various cancers [21–23]. TGF-β plays a dual role in cancer progression: it exerts an inhibitory effect in the early stages of tumor development but promotes progression, migration, invasion, angiogenesis, and metastatic propagation in the later stages [24, 25]. In PC, TGF-β has been shown to provide an initiation signal for epithelial-mesenchymal transition (EMT), leading to the EMT-inducing transcriptional factors upregulation [26]. TGF-β also affects nuclear accumulation of the nuclear factor-kappa B (NF-κB), which leads to morphological changes in PC cells to the mesenchymal phenotype [27, 28]. Further, a study on NEPC reported TGF-β signaling promoted the invasiveness of PC cells, suggesting a strong link between TGF-β signaling and NEPC development [29–32]. Therefore, identifying regulators of TGF-β signaling in PC progression can help understand the development of highly aggressive PC.

In this study, we aimed to identify the *ZNF507* function in PC and metastatic aggressive PC. We found that *ZNF507* shows greater expression in the NE-like regions of PC and brain-metastasized PC cells. We demonstrate that *ZNF507* upregulates genes for TGF-β signaling, including TGF-β receptor 1 (TGFBR1), mitogen-activated protein kinase 8 (MAP3K8), and the Paired Basic Amino Acid Cleaving Enzyme (FURIN). *ZNF507* exacerbates the progression of PC, which results in aggressive metastatic features. Our findings suggest that *ZNF507* is a regulator of TGF-β signaling in the progression of PC to malignancy, and shows potential for the attractive marker of advanced metastatic PC.

## Methods

### Patient samples

The PC tissue specimens were obtained from the Korea Biobank Network-KNUH, after obtaining the consent of the donors (KNUMC 2016-05-021). All experimental processes utilizing human tissues were performed at Daegu Gyeongbuk Institute of Science & Technology (DGIST) with IRB approval (DGIST-190,319-BR-006-02).

### Cell culture

Human prostate RWPE1 cells were cultured in keratinocyte serum-free medium (K-SFM) (#17005-042, Gibco, Grand Island, USA). The human PC cell lines PC3, PC3M, and 22Rv1 were cultured in RPMI (SH30027, Hyclone, MA, USA), and DU145 cells were cultured in DMEM (SH30243, Hyclone). All cells were cultured in medium containing 10 % fetal bovine serum (Hyclone) and 1 % penicillin/streptomycin (Gibco).

### Lentiviral transduction and cell transfection

pLKO.1-scramble and shZNF507 (Sigma, MO, USA) lentivirus containing supernatants were prepared through HEK293T mediated viral particle production. DU145 and 22Rv1 cells were treated with the lentivirus containing supernatants and 8 µg/ml polybrene (Sigma). After 48 h of incubation, the lentivirus-infected PC cells were selected by treatment with 1.5 µg/ml puromycin for 7 days (Invivogen, Hong Kong).

The pcDNA3.1 and pcDNA3.1-ZNF507 plasmid vectors were transfected to DU145 cells using Lipofectamine2000 (Thermo Fisher, MA, USA) following the manufacturer’s instruction. After 7 days of G418 (1.5 mg/ml, Sigma) selection, stable cell lines were established by confirming the ZNF507 expression. The same concentration of puromycin or G418 was treated every 3 days.

### Cell proliferation assay

96-well seeded DU145 and 22Rv1 cells were treated with 10 µl of Cell Counting Kit-8 solution (CCK-8) (Dojindo, MD, USA) for 1 h before conducting the measurements. The plates were read at an optical density of 450 nm using a SpectraMaxiD3 spectrophotometer (Molecular Devices, CA, USA).

Proliferating cell nuclear antigen (PCNA) in DU145 and 22Rv1 cells were stained with primary antibody against PCNA (ab18197; Abcam, Cambridge, UK) followed by Alexa594 secondary antibody (A11037; Thermo Fisher) staining. DAPI-containing Vectashield (Vector Laboratories, CA, USA) were used for mounting and microscopic imaging (CKX53, Olympus, Tokyo, Japan) with U-HGLHPS illumination system (Olympus) and eXcope X7M camera (DIXI Science, Daejeon, Korea).

### Anchorage-independent colony formation

DU145 and 22Rv1 cells were suspended in basal medium Eagle (BME), mixed with 0.3 % agar (top agar), and seeded over a 0.5 % agar (bottom agar) base layer of 6-well plate. After 3 weeks incubation, the number and diameter of colonies were measured. The colony was defined as a cluster of 50 or more cells.

### Invasion and migration assay

Matrigel (BD BioScience, CA, USA) pre-coated 8 μm-pore trans-well chambers (Corning, NY, USA) were loaded with 5 × 10^4^ cells in 200 µl of serum-free media, and the lower well was filled with 600 µl of media for 48 h incubation. After formaldehyde fixation, followed by 0.05 % crystal violet (Sigma) staining, images were captured. The method for performing the migration assay was the same as for the invasion assay, except for the trans-well Matrigel pre-coating step.

### Cell cycle and apoptosis analyses

DU145 and 22Rv1 cells were fixed with 100 % ethanol for 1 h and incubated with 500 µl of PI solution (Invitrogen, CA, USA) in the dark for 15 min. The DNA content of the cells was analyzed using a BD-Accur-C6 flow cytometer (BD Biosciences). For each sample, at least 20,000 events were acquired. For the apoptosis assay, the Dead-Cell-Apoptosis-Kit with AnnexinV-Alexa-Fluor™488 & Propidium Iodide (PI) kit (Invitrogen) was used as per the manufacturer’s instructions. Data from cells was acquired by using the BD-Accuri-C6 flow cytometer (BD Biosciences), and 10,000 events per sample were acquired.

### Quantitative reverse transcription PCR (qRT-PCR)

cDNA was synthesized from harvested RNA by using the MiniBEST Universal RNA extraction kit (cat#9767; Takara, Shiga, Japan), as per the manufacturer’s instructions. qRT-PCR analysis was conducted using the StepOnePlus RT-PCR system (Thermo Fisher) with TB Green Premix EX Taq (Takara). The relative mRNA expression scores were calculated following the 2^−ΔΔCt^ method [33]. The primers used in qRT-PCR analysis are listed in the Table S1.

### Immunoblot

Proteins from cell lysates or nuclear cytosol fractionized samples extracted by nuclear cytosol fractionization kit (Thermo Fisher) separated using 4–15 % or 4–20 % Mini-PROTEAN® TGX™ Precast Protein Gels (Bio-rad) were transferred to nitrocellulose membranes, which were subsequently blocked for 2 h with 5 % skim milk or 5 % BSA. Primary antibodies (Table S2) diluted in the blocking solution were incubated overnight at 4℃. After thrice Tris-buffered saline with 0.05 % Tween-20 washing, and secondary antibodies incubation for 2 h at room temperature, the membrane was developed by using the Clarity Western ECL substrate (Bio-rad), and visualized using the ImageQuant LAS500 system (GE Healthcare, Uppsala, Sweden).

### Dual-luciferase reporter assay

The human DNA sequences for the *TGFBR1* promoter [-1378 to + 75 bp (bp)], and the *MAP3K8* promoter [-1441 to 0 bp] were cloned into the pGL3-basic vector. DU145 cells (1.5 × 10^4^ cells per well) in 96-well plates were co-transfected with the pGL3- control, pcDNA3.1, or the pcDNA3.1-ZNF507 plasmids. After 48 h, luciferase activity was measured using the SpectraMaxiD3 spectrophotometer (Molecular Devices). Firefly activity was normalized to renilla activity.

### Chromatin immunoprecipitation (ChIP)

ChIP assays were conducted using the EZ-Magna ChIP kit (Sigma) as per the manufacturer’s instructions. The following antibodies were used: ZNF507 (A303-274a; Bethyl Laboratories, TX, USA) and rabbit IgG (ab46540; Abcam). ChIP qRT-PCR analysis was performed on the purified DNA using four site-specific primer sets in the *TGFBR1* promoter region (R1, -1541 to -1456 bp; R2, -1439 to -1298 bp; R3, -747 to -647 bp; R4, -396 to -309 bp) and three site-specific primer sets in the *MAP3K8* promoter region (R1, -1405 to -1324 bp; R2, -1166 to -1079 bp; R3, -296 to -225 bp). The list of primers used in this study are described in Table S1.

### Xenograft

All animal experiments were conducted with the permission and guidelines from the DGIST Laboratory Animal Resource Center. Five-week-old male BALB/c nude mice were subcutaneously injected with 200 µl PBS containing 6 × 10^6^ scramble, shZNF507, pcDNA3.1, or pcDNA3.1-ZNF507 DU145 cells. The injections were administered to each side of the dorsal region. The tumor volume was calculated by blind grouping for every 3 days until sampling. On the last day, the mice were sacrificed and the tumors were randomly excised for western blot or histological analysis.

### *In vivo* metastasis analysis

Five-week-old male BALB/c nude mice were intravenously injected with 200 µl PBS containing 1 × 10^6^ scrambled or shZNF507 treated DU145 cells. Five weeks after injection, lungs and livers of all mice were sampled for the histological analysis.

### Immunohistochemistry and immunofluorescence

Samples of benign hyperplasia and PC patient and xenograft tumors were fixed in 10 % formaldehyde, embedded in paraffin, and sectioned. Immunohistochemical staining of ZNF507 (ab85672; Abcam) was performed using the ZytoChemPlus HRP Kit (Zytomed, Berlin, Germany) as per the manufacturer’s instructions. For immunofluorescent staining, primary antibodies (Table S2) were incubated after 1 % BSA in PBST blocking. Alexa488 (A11001; Thermo Fisher) and Alexa594 (A11037; Thermo Fisher) secondary antibodies were incubated for 2 h in a dark chamber followed by DAPI-containing Vectashield (Vector Laboratories) mounting.

### Microarray and bioinformatics

GeneChip analysis was performed with scrambled shRNA or shZNF507 treated PC cells. Expression data was analyzed using ExDEGA (EBIOGEN Inc., Seoul, Korea), gene ontology (GO) analysis, and gene set enrichment analysis (GSEA). The related pathway analyses were performed using the Molecular Signature Database (MSigDB) and the Kyoto Encyclopedia of Genes and Genomes (KEGG) database.

The expression profiles of *ZNF507*, *TGFBR1*, *MAP3K8*, and *FURIN* were analyzed using cancer datasets from various databases, including Gene Expression database of Normal and Tumor tissues (GENT2), The European Bioinformatics Institute (EMBL-EBI), Gene Expression Omnibus (GEO), cBioPortal for cancer genomics, and GEPIA.

### Statistical analysis

All data were analyzed in triplicate from at least three independent experiments, and the results summarized as mean (SD). Mean differences were assessed by an analysis of variance (ANOVA) using GraphPad Prism 5 (GraphPad, San Diego, CA, USA), and a *p*-value ≤ 0.05 was considered significant.

## Results

### Elevated *ZNF507* as a prognostic factor in highly graded PC patients

**Fig. 1:**
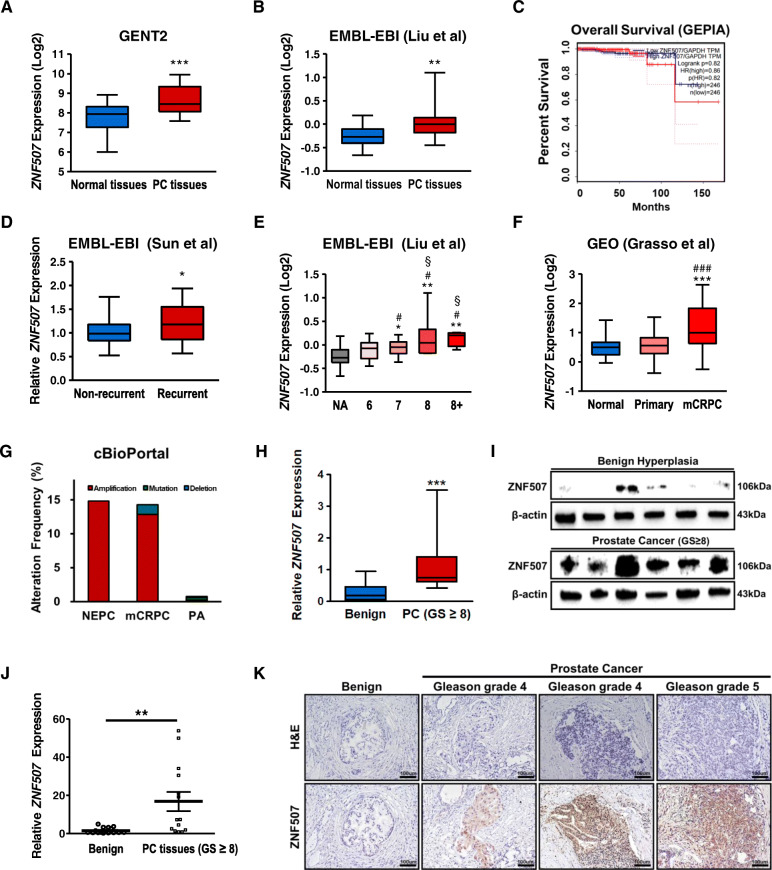
Increased ZNF507 expression in various PC databases and PC tissues. **A** *ZNF507* expression data in normal (*n* = 42) and PC tissues (*n* = 277) as retrieved from the GENT2 database. **B** *ZNF507* expression data in normal (*n* = 13) and PC tissues (*n* = 44) as retrieved from the EMBL-EBI database (Liu et al.). **C** The survival rate of PC patients compared by low-ZNF507 (*n* = 246) expressed and high-ZNF507 (*n* = 246) expressed groups from the GEPIA database. TPM; Transcripts per million, HR; hazard ratio. The median value was used for the group classification. *p* = 0.82, HR = 0.86. **D** Relative *ZNF507* expression in non-recurrent (primary) PC (*n* = 37) and recurrent PC tissues (*n* = 45) as retrieved from the EMBL-EBI database (Sun et al.). **E** *ZNF507* expression in the normal and Gleason graded PC tissues as retrieved from the EMBL-EBI database (Liu et al.) (NA, *n* = 13; grade 6, *n* = 13; grade 7, *n* = 16; grade 8, *n* = 10; grade 8+, *n* = 5; *, compared to NA group; #, compared to grade 6 group; §, compared to grade 7 group). **F** *ZNF507* expression in normal (*n* = 28), primary PC (*n* = 59), and mCRPC (*n* = 34) as retrieved from the GEO database (*, compared to normal group; #, compared to primary PC group). **G** Genomic alteration frequency of ZNF507 in NEPC, mCRPC, and PA as retrieved from the cBioPortal database. **H** Average of the relative ZNF507 expression from benign hyperplasia (*n* = 6) and Gleason score 8 patient tissues (*n* = 15) specimens compared to its related β-actin intensity. **I** Representative western blot data showing ZNF507 expression from benign hyperplasia and Gleason score over 8 PC patient tissues. **J** Relative mRNA expression of *ZNF507* from benign hyperplasia (*n* = 16) and Gleason score 8 PC tissues from patients (*n* = 14). **K** Representative IHC staining of ZNF507 in benign hyperplasia and Gleason grade 4 and 5 PC tumor tissues (Scale bar = 100 μm). The data are presented as the Means ± SD from three independent experiments. *p < 0.05, **p < 0.01, ***p < 0.001 versus normal control

We first analyzed the *ZNF507* expression in PC tissues using data from various databases. GENT2 and EMBL-EBI data analyses revealed significant *ZNF507* upregulation in PC tissues (Fig. 1A, B). Interestingly, since there was no significant survival rate difference in prostate adenocarcinoma (PA) patients who showed high or low *ZNF507* expression, considerably higher ZNF507 level was observed in recurrent PC tumors (Fig. 1C, D). Similarly, *ZNF507* strongly expressed in high Gleason score PC in EMBL-EBI data (Fig. 1E). GEO database results showed increased *ZNF507* in mCRPC tumors compared with that in normal or primary PC tissues (Fig. 1F). The PC type alteration frequency data in cBioPortal indicated the *ZNF507* alteration frequency showed 14.81 % amplification in NEPC, 12.86 % amplification and 1.43 % mutation in mCRPC, and only 0.21 % amplification, 0.38 % mutation, and 0.178 % deletion in PA (Fig. 1G). To confirm our findings from the databases, we evaluated the *ZNF507* expression in specimens from benign hyperplasia and high Gleason score (over 8) PC tissues and checked significantly elevated *ZNF507* in aggressive PC tissues (Fig. 1H-J). As there was a remarkable increase in *ZNF507* in mCRPC and an elevated alteration frequency for NEPC or mCRPC in the database, we examined the *ZNF507* expression with the neuroendocrine marker NeuroD1 in benign hyperplasia and highly graded PC tissues. The results showed both *ZNF507* and NeuroD1 were highly expressed in the malignant regions of the PC tissues (Fig. 1K, Fig.S1).

### *ZNF507* knockdown suppresses the growth, proliferation, and metastatic properties of PC cells

**Fig. 2:**
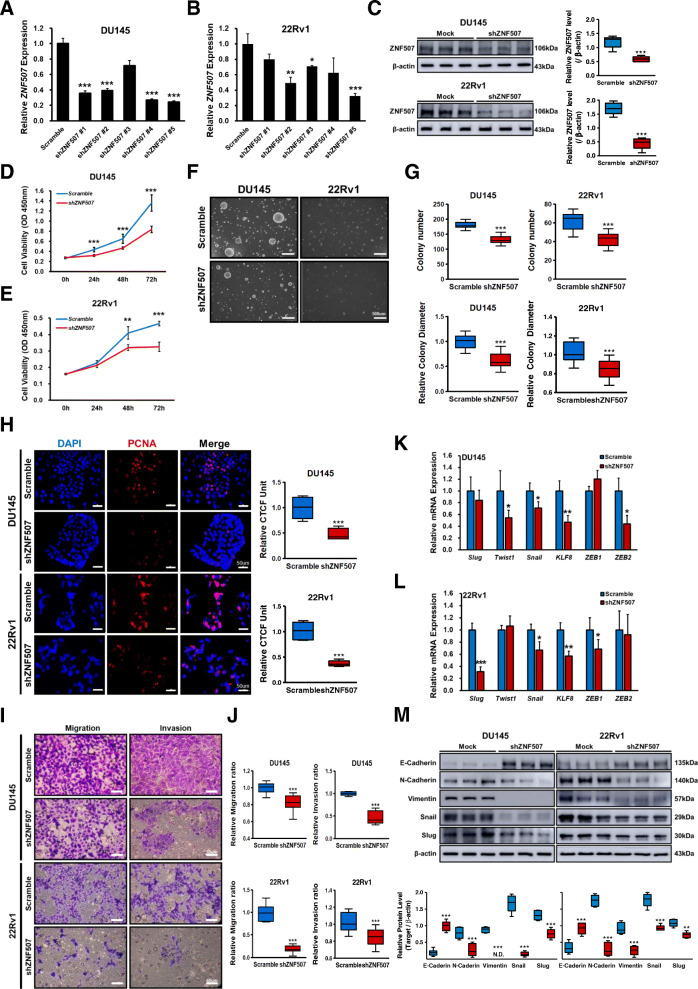
Decreased cancer properties, including cell proliferation, colony formation, and metastasis from ZNF507 knockdown in PC cells. **A**, **B** Relative *ZNF507* mRNA levels from shRNA-mediated knockdown compared with the scramble control in DU145 and 22Rv1 cells. Each cell lines were prepared for three independent experiments with at least three samples per group. **C** Representative protein expression data assessed by western blot for ZNF507 in scramble or shZNF507 DU145 or 22Rv1 cells; shZNF507 #5 treated DU145 and 22Rv1 cells. **D**, **E** CCK-8 proliferation assay of the scramble or shZNF507 DU145 or 22Rv1 cells. The optical density (OD) value at 450 nm was determined. Each experiment consists of six samples from each group of cells were performed. Three independent experiments were performed. **F** Colony formation of the shZNF507 treated DU145 or 22Rv1 cells performed by soft agar assay. **G** Statistical analysis of colony number and relative diameter analyzed from the colony formation assay data. Each experiment consists of four samples from the same group of cells were measured and the three independent experiments were conducted. **H** PCNA staining of the scramble or shZNF507 DU145 or 22Rv1 cells performed by immunocytochemistry (Scale bar = 50 μm). The graph in the right panel presents relative corrected total cell fluorescence (CTCF). At least 4 pictures from each sample were taken and the CTCF were calculated. **I** Representative images of the invasion and migration assay performed with the scramble or shZNF507 DU145 or 22Rv1 cells using trans-well plate (Scale bar = 50 μm). **J** Relative ratio of migration and invasion of the data from the transwell migration and invasion assay. At least four pictures from each sample were taken and the three independent experiments were performed. **K**, **L** Relative mRNA expression of *Slug*, *Twist1*, *Snail*, *KLF8*, *ZEB1*, and *ZEB2* in the scramble or shZNF507 DU145 or 22Rv1 cells analyzed by qRT-PCR. *GAPDH* was used as a normalization control. The three independent experiments consist of at least four samples per group were performed. **M** Representative images of protein expression data of E-cadherin, N-cadherin, Vimentin, Snail, and Slug in the scramble or shZNF507 DU145 or 22Rv1 cells assessed by western blot. β-actin was used as an endogenous control. The data are presented as the Means ± SD from three independent experiments. *p < 0.05, **p < 0.01, ***p < 0.001 versus scramble control

Next, we analyzed *ZNF507* levels in human prostate and various PC cell lines. We selected two PC cell lines, DU145 and 22Rv1, displayed high *ZNF507* expression (Fig.S2A, B). Following the *ZNF507* knockdown, the shZNF507 #2 and #5 of both cell lines was selected based on their lowered mRNA and protein levels; the shZNF507 #5 (Fig. 2C) treated DU145 and 22Rv1 cells displayed nearly halved or lower ZNF507 expression, whereas those of the shZNF507 #2 (Fig.S3A) displayed nearly 30 % decrease in ZNF507 protein expression (Fig. 2A-C, Fig.S3A). We also confirmed that ZNF507 expressed in both nuclear and cytosolic area and knockdown of the gene was efficiently performed in both area (Fig.S2C, D). The knockdown of *ZNF507* in PC cells induced a decrease in the proliferation rate (Fig. 2D, E). Further, colony formation ability was significantly repressed by the *ZNF507* knockdown (Fig. 2F, G, Fig.S3C). The cell proliferative marker PCNA intensity was significantly reduced in the *ZNF507* knockdown cells (Fig. 2H, Fig.S3B).

In the invasion and migration assay, decline in the migration and invasion ratio by *ZNF507* knockdown in both PC cell lines were observed (Fig. 2I, J, Fig.S3D). We next confirmed the changes of epithelial-mesenchymal transition (EMT) markers, Twist1, Snail1, *KLF8*, and *ZEB2* in DU145, and Slug, Snail, *KLF8*, and *ZEB1* in 22Rv1 by ZNF507 knockdown (Fig. 2K, L). The anti-metastatic *ZNF507* knockdown effect was checked by protein EMT markers expression; elevated E-cadherin and decreased N-cadherin, Vimentin, Snail, and Slug in both DU145 and 22Rv1 cells, indicating *ZNF507* may affect the growth, proliferation, and metastatic properties of PC cells (Fig. 2M, Fig.S3E).

### *ZNF507* knockdown leads to cell cycle alteration and promotes apoptosis

**Fig. 3:**
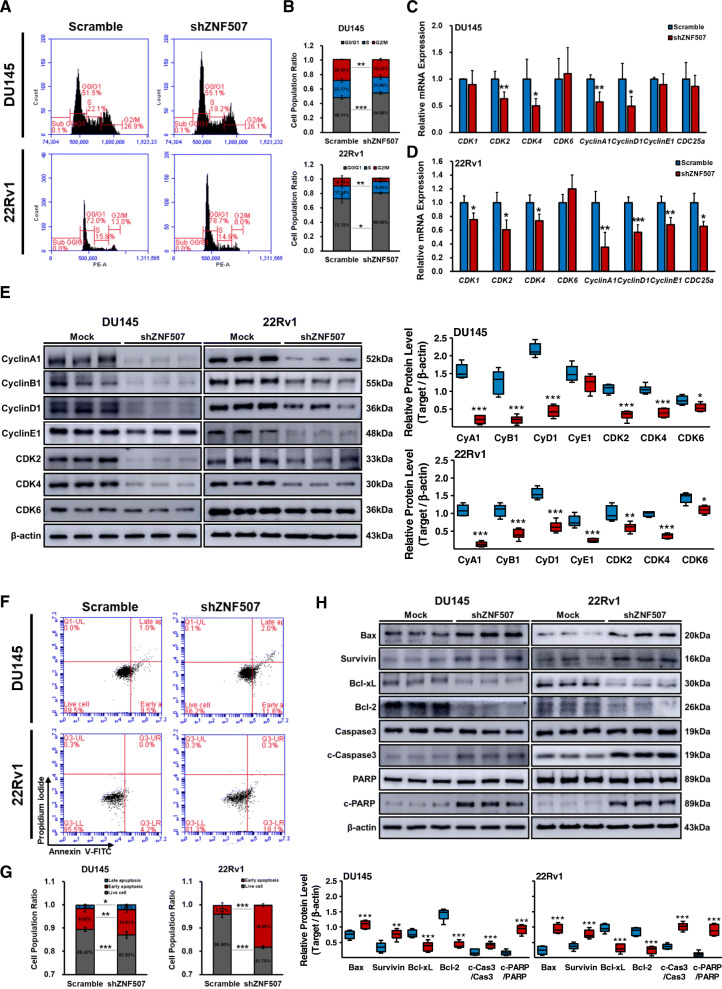
ZNF507 knockdown induce cell cycle arrest and apoptosis in PC cells. **A** Cell cycle analysis was performed with scramble or shZNF507 DU145 or 22Rv1 cells by propidium iodide (PI) staining. The proportion of cells in each cycle was measured. **B** Ratio of cell cycle population from cell cycle analysis. Three independent experiments were performed and at least four samples per group were measured in each experiment. **C**, **D** Relative mRNA expression of *CDK1*, *CDK2*, *CDK4*, *CDK6*, *CyclinA1*, *CyclinD1*, *CyclinE1*, and *CDC25a* in the scramble or shZNF507 DU145 or 22Rv1 cells assessed by qRT-PCR. *GAPDH* was used as normalization control. The data are presented as the Means ± SD from three independent experiments. **E** Representative images of the protein expression data of CyclinA1, CyclinB1, CyclinD1, CyclinE1, CDK2, CDK4, and CDK6 in the scramble or shZNF507 DU145 or 22Rv1 cells assessed by western blot. β-actin was used as an endogenous control. **F** Apoptosis analysis conducted by PI-Annexin V-FITC staining in the scramble or shZNF507 DU145 or 22Rv1 cells. **G** Graph of cell population ratio from the apoptosis analysis of the scramble or shZNF507 DU145 or 22Rv1 cells. Three independent experiments were performed and at least 4 samples per group were measured in each experiment. **H** Representative images of the protein expression data of Bax, Survivin, Bcl-xL, Bcl-2, Caspase3, cleaved-Caspase3, PARP, and cleaved-PARP in the scramble or shZNF507 DU145 or 22Rv1 cells assessed by western blot. β-actin was used as an endogenous control. For all the data, at least three independent experiments were performed. *p < 0.05, **p < 0.01, ***p < 0.001 versus scramble control

As cell cycle is essential for proper cell proliferation [34], we examined cell cycle via flow cytometry. shZNF507 treated DU145 and 22Rv1 cells displayed significantly elevated G0/G1 phase cell population with decline in G2/M phase population, indicating the cell cycle phases length change by reduced *ZNF507* (Fig. 3A, B, Fig.S3F). To further investigate the relevance, we examined the cell cycle checkpoint markers expression, *CDK1*, *CDK2*, *CDK4*, *CDK6*, CyclinA1, CyclinD1, CyclinE1, and *CDC25A*. As a result, the majority of the checkpoint markers were altered in both shZNF507 treated PC cell lines (Fig. 3C, D). Further, the protein expression of CyclinA1, CyclinB1, CyclinD1, CyclinE1, CDK2, and CDK4 was substantially diminished in shZNF507 treated PC cells, while there was no significant change in CDK6 (Fig. 3E, Fig.S3G).

Next, we evaluated the cell survival rate induced by *ZNF507* knockdown using flow cytometry-based assays. We found that the ratio of early and late apoptosis was significantly increased in shZNF507 treated DU145 cells, and only early apoptosis was increased in shZNF507 treated 22Rv1 cells, with almost no detection of cells in late apoptosis in 22Rv1 cells (Fig. 3F, G, Fig.S3H). Interestingly, we found that the ratio of apoptotic population in DU145 and 22Rv1 was differently altered depending on the efficiency of *ZNF507* knockdown. The association with increased apoptosis due to *ZNF507* knockdown was confirmed from the elevated Bax, Survivin, cleaved-Caspase3, and cleaved-PARP, along with lowered Bcl-xL and Bcl-2 levels in shZNF507 treated PC cells (Fig. 3H, Fig.S3I). We next checked whether the changes in these factors were relevant in ZNF507 overexpression DU145 cells (Fig. 5G, Fig.S6). Taken together, we confirmed that the PC growth and proliferation change by *ZNF507* knockdown were emerged with cell cycle stage length change and apoptotic death.

### *ZNF507* knockdown reduces TGF-β signaling in PC cells

**Fig. 4:**
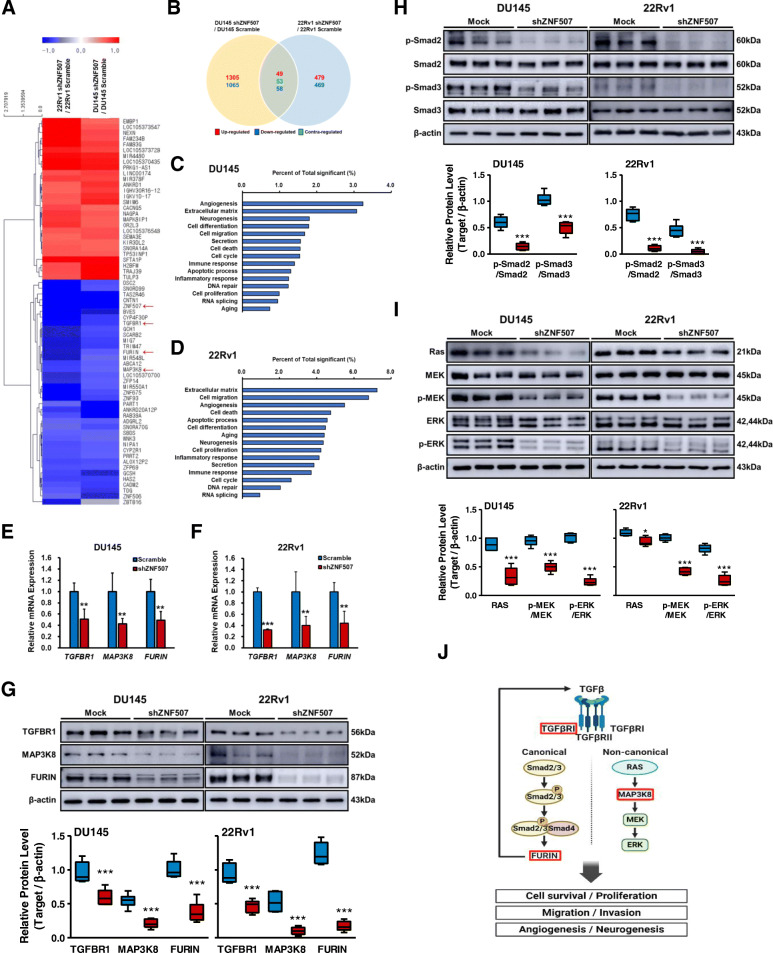
Knockdown of ZNF507 in PC cells displays altered TGF-β signaling. **A** Heat map of differentially expressed gene values in shZNF507 cells compared with their respective scramble groups, as performed by microarray analysis. Red arrows indicate the state of *ZNF507*, *TGFBR1*, *MAP3K8*, and *FURIN*. **B** Venn diagram describing the number of differentially expressed genes in DU145 and 22Rv1 experiments. Font in red indicates the number of up-regulated genes, blue indicates the number of down-regulated genes, and green indicates the number of inversely-regulated genes in both groups. **C**, **D** KEGG pathway enrichment analysis in the shZNF507 DU145 or 22Rv1 cells compared with their respective scramble groups. **E**, **F** Relative *TGFBR1*, *MAP3K8*, and *FURIN* mRNA expression assessed by qRT-PCR analysis of the shZNF507 DU145 or 22Rv1 cells compared with their respective scramble cells. *GAPDH* was used as an endogenous normalization control. The data are presented as the Means ± SD from three independent experiments. **G** Representative images of the protein expression data of TGFBR1, MAP3K8, and FURIN detected by western blot in the scramble and shZNF507 #5 DU145 or 22Rv1 cells. **H** Representative images of the protein expression data of canonical TGF-β signal proteins, Smad2, and Smad3 with their phosphorylated forms, assessed by western blot. β-actin was used as a normalization control. **I** Representative images of the protein expression data of non-canonical TGF-β signal proteins, RAS, MEK, phosphorylated-MEK, ERK, and phosphorylated-ERK, examined by western blot. β-actin was used as a normalization control. **J** Schematic of TGF-β signal pathway in PC and related factors (red framed). For all the data, at least three independent experiments were performed. **p< 0.01, ***p < 0.001 versus scramble control

To profile the related transcriptional changes underlying *ZNF507* in PC, we performed a microarray. A total of 49 co-upregulated and 58 co-downregulated genes were profiled (Fig. 4A, B), and a GO based analysis revealed the several gene clusters enrichment related to various cancer properties, including angiogenesis, differentiation, migration, cell death, apoptotic process, and neurogenesis (Fig. 4C, D, Table S3). Considering its multiple tumor-promoting effects in various cancers [35], we focused on TGF-β signaling-related genes, namely *TGFBR1*, *MAP3K8*, and *FURIN* (Fig. 4A, red arrow). *TGFBR1* encodes the TGF-β receptor 1 subunit of the receptor complex and plays a crucial role in downstream Smad-dependent canonical and Smad-independent non-canonical signaling [25, 26]. *MAP3K8* is a mediator of the non-canonical TGF-β signaling pathway, proposed to be a proto-oncogene that activates the downstream MEK-ERK pathway in various cancers [36–38]. The *FURIN* is regulated by Smad-dependent canonical TGF-β signaling, and it is a proprotein convertase which plays a role in the cleavage of pro-TGF-β to its mature form, eventually stimulating the positive feedback loop of TGF-β signaling in cancers [39–41]. After confirming TGFBR1, MAP3K8, FURIN alteration by ZNF507 knockdown in PC cells (Fig. 4E-G, Fig.S4a), we examined the TGF-β downstream signals, and verified the diminished signal cascade activation (Fig. 4H, I, Fig.S4B, C). Expression of other several genes of GO analysis were confirmed by qRT-PCR in shZNF507 and ZNF507 overexpression cells (Fig.S5). A detailed explanation of TGF-β and its related signal pathway is provided in the schematic presented in Fig. 4J.

### Upregulation of TGFBR1, MAP3K8, and FURIN by ZNF507 in PC

**Fig. 5:**
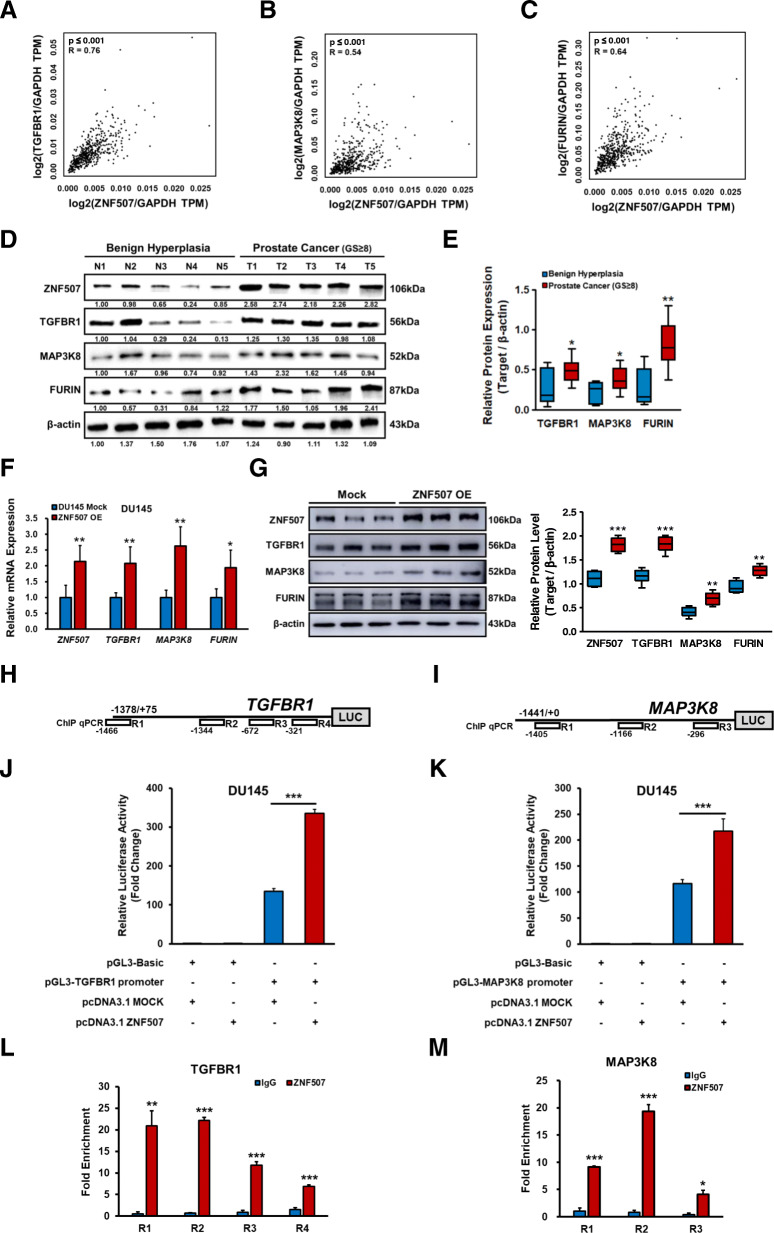
ZNF507 as an activator of TGFBR1, MAP3K8, and FURIN transcription, and their positive correlation in PC. **A** Spearman correlation analysis of ZNF507 and TGFBR1 levels compared to the Gapdh levels in PC samples based on the GEPIA database (TPM, Transcripts per Kilobase Million, *n* = 472). **B** Spearman correlation analysis of ZNF507 and MAP3K8 expression normalized to the Gapdh levels in PC based on the GEPIA database (*n* = 472). **C** Spearman correlation analysis of ZNF507 and FURIN normalized to the Gapdh levels in PC from the GEPIA database (*n* = 472). **D** Representative protein expression data of ZNF507, TGFBR1, MAP3K8, and FURIN in the benign hyperplasia and PC tissue (Gleason score over 8) specimens assessed by western blot. β-actin was used as a normalization control. **E** Average of relative protein expression of ZNF507, TGFBR1, MAP3K8, and FURIN compared with the intensity of β-actin in the benign hyperplasia (*n* = 6) and PC tissue (Gleason score over 8m *n* = 15) specimens. Three independent experiments were performed and the average rate of each sample were analyzed. **F** Relative mRNA expression of *ZNF507*, *TGFBR1*, *MAP3K8*, and *FURIN* determined by qRT-PCR analysis of the ZNF507 overexpression (ZNF507 OE) DU145 cells compared to the mock control.**G** Representative images of the protein levels of ZNF507, TGFBR1, MAP3K8, and FURIN in the mock and ZNF507 OE DU145 cells examined by western blot. β-actin was used as a normalization control. **H**, **I** Schematics of TGFBR1 and MAP3K8 promoter regions and related four sets of ChIP qRT-PCR primers. **J** Dual reporter luciferase assay conducted with pGL3-Basic or pGL3 containing TGFBR1 promoter vectors and pcDNA3.1-Mock or pcDNA3.1-ZNF507 vectors. **K** Dual reporter luciferase assay conducted with pGL3-Basic or pGL3 containing MAP3K8 promoter vectors and pcDNA3.1-Mock or pcDNA3.1-ZNF507 vectors. **L** qRT-PCR analysis of TGFBR1 promoter regions conducted with 4 sets of specific primers after a ChIP assay with DU145 cells, with anti-ZNF507 antibody or the IgG isotype control. The results presented are fold enrichments relative to IgG control. **M** qRT-PCR analysis of MAP3K8 promoter regions conducted with 3 sets of specific primers after a ChIP assay in DU145 cells using an anti-ZNF507 antibody to query binding, with IgG used as an antibody control. The results presented are fold enrichment relative to the IgG control. Data are presented as the Means ± SD from three independent experiments including at least 3 independents samples. *p < 0.05, **p < 0.01, ***p < 0.001 versus normal tissue or Mock control

To evaluate the relationship between ZNF507 and TGF-β signal factors, we conducted a correlation analysis using the GEPIA database. The spearman correlation (R) score between ZNF507 and TGFBR1 in PC was 0.76 (*p* < 0.001), indicating strong positive correlation (Fig. 5A). The score between ZNF507 and MAP3K8 (0.54, *p* < 0.001) and FURIN (0.64, *p* < 0.001) was also analyzed (Fig. 5B, C). To further determine this in our patient PC tissues, we assessed TGFBR1, MAP3K8, and FURIN levels and confirmed the elevated expression in the high Gleason scored PC tissues compared with benign hyperplasia tissues (Fig. 5D-F). We next checked whether the changes in these factors were relevant in ZNF507 overexpression DU145 cells (Fig. 5G, Fig.S6). We additionally investigated the transcriptional activity of *TGFBR1* and *MAP3K8* promoters regulated by ZNF507; since *FURIN* was already known to be regulated by Smad2/3 activation, we did not check its transcriptional activity [41]. We assessed the promoter activity of *TGFBR1* and *MAP3K8* by ZNF507 overexpression in DU145 cells and confirmed significant elevation of both genes (Fig. 5J, K). Furthermore, using ChIP qRT-PCR analysis with four site-specific primer sets of *TGFBR1* and three primer sets of *MAP3K8* promoter regions, based on released data of ZNF507 ChIP-seq on human MCF-7 cancer cells (NCBI, ENCSR4190DQ), we determined the putative ZNF507-binding region in the promoter of both genes (Fig.S7, Fig. 5H-M). This suggests ZNF507 is a potential transcriptional regulator of *TGFBR1* and *MAP3K8*.

### ZNF507 depletion suppresses tumor growth and metastasis *in vivo* and attenuates NEPC-like phenotype by TGF-β signaling inhibition

**Fig. 6:**
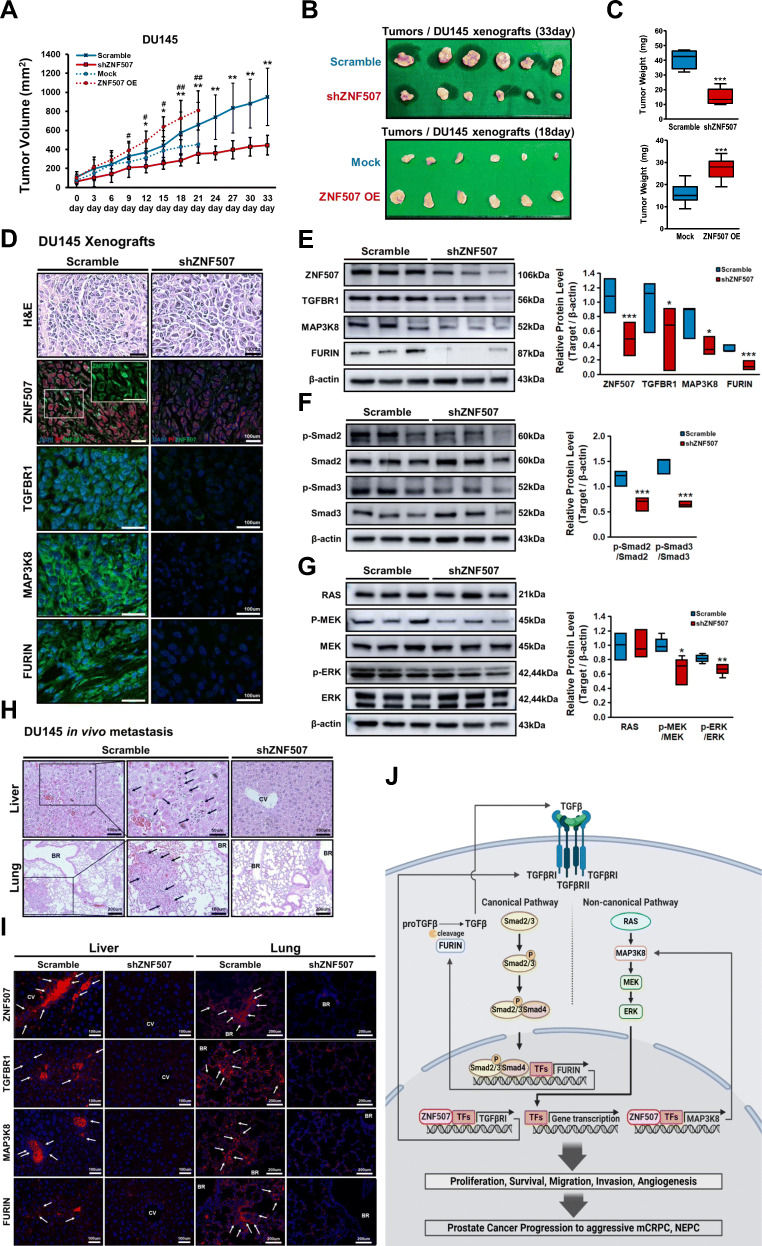
Knockdown of ZNF507 leads to altered tumor growth *in vivo* and diminish NEPC-like phenotypes by TGF-β signaling inhibition. **A** Growth curve of the tumor formation after the scramble, shZNF507, Mock, or ZNF507OE DU145 cells were xenografted into Balb/C nude mice (*n* = 6 per each set group). For each group, three tumor samples were used for the Western blot analysis, while other three samples were used for the IF. **B** Tumors from scramble and shZNF507 group were extracted on the last day (day 33) and Mock and ZNF507 OE group were extracted on day 18 of the xenograft experiments conducted through injection. **C** Graph showing the average weight of the scramble and shZNF507 xenografted tumors (*n* = 6 per each group). **D** Histological analysis of tumors from the scramble and shZNF507 xenograft. Representative data of H&E staining and immune staining against ZNF507 (with PI), TGFBR1, MAP3K8, and FURIN in tumors, performed by immunofluorescence (Scale bar = 100 μm). **E** Representative protein expression data of ZNF507, TGFBR1, MAP3K8, and FURIN in the xenografted tissues as determined by western blot analysis. β-actin served as a loading control. Right panel shows the average of the relative protein levels normalized to the β-actin. At least three samples per group were used for each experiment and the three independent experiments were performed and analyzed. **F** Representative protein expression data of canonical TGF-β signal proteins, Smad2, Smad3, and their phosphorylated forms, assessed by western blot. β-actin was used as a normalization control. At least three samples per group were used for each experiment and the three independent experiments were performed and analyzed. **G** Representative protein expression levels of non-canonical TGF-β signal proteins, RAS, MEK, and p-MEK, ERK, and p-ERK, examined by western blot. β-actin was used as a normalization control. At least three samples per group were used for each experiment and the three independent experiments were performed and analyzed. **H** Representative data of H&E staining of the liver and lung from *in vivo* metastasis assay. **I** Representative images of the immune staining against ZNF507, TGFBR1, MAP3K8, and FURIN in liver and lung from *in vivo* metastasis assay, performed by immunofluorescence. **J** Schematics presenting the biological role of ZNF507 in TGF-β signaling, as a transcriptional activator, and related pathway in the progression of PC to its aggressive mCRPC and NEPC state. Data are presented as the Means ± SD from three independent experiments. #*p* < 0.05, ##*p* < 0.01, Mock versus the ZNF507 OE xenograft tissue and *p < 0.05, **p < 0.01, ***p < 0.001 shZNF507 versus the scramble xenograft tissue

To validate the promoting effect of ZNF507 in PC progression, we conducted an *in vivo* DU145 xenograft (the 22Rv1 was excluded due to its slow growth rate). The growth rate, volume, and weight of the ZNF507 knockdown xenografted tumors declined significantly, while ZNF507 OE tumors increased in faster rate (Fig. 6A-C, Fig.S8A, Fig.S11A, B). Fluorescent staining of tumors showed relatively elevated Ki67 levels with lowered cleaved-Caspase3 expression in scramble xenografted tumors compared to ZNF507 knockdown xenografted tumors (Fig.S8B). Further, we detected intensified expression pattern for ZNF507, TGFBR1, MAP3K8, and FURIN in the scramble xenografted tumors (Fig. 6D, Fig.S8C). We additionally confirmed the altered activation of the downstream TGF-β signaling cascade (Fig. 6E-G, Fig.S8D-F, Fig.S11C-E). In the analogous staining of the neuroendocrinal markers, NeuroD1 and Synaptophysin, we detected a pattern similar to that of ZNF507 and TGF-β cascades (Fig.S9, Fig.S11F). This was consistent with the staining pattern of ZNF507 overexpression cells (Fig.S10). Finally, we conducted *in vivo* metastasis analysis, and identified the ZNF507 knockdown remarkably suppressed the metastasis of DU145 cells in the lung and liver with the decrease of TGFBR1, MAP3K8, and FURIN (Fig. 6H, I).

## Discussion

Since castration-resistant metastatic features remain a major obstacle for PC treatment, identifying novel marker for metastatic aggressive PC is essential for improving PC treatment [3, 6, 42]. In the current study, we identified the biological significance of ZNF507 in the progression of PC to aggressive phenotype by regulating TGF-β signaling (Fig. 6 H).

The data retrieved from PC databases indicated prominent ZNF507 expression in high-grade PCs. Our findings on ZNF507 pattern are consistent with ZNF507 expression data in Fig. S2: higher ZNF507 in DU145 and 22Rv1 cells, which were derived from brain-metastatic cells (DU145) and propagated xenograft after castration-induced regression (22Rv1) [43, 44]. As the ZNF507 expression was lowered in bone metastatic PC cell line PC3M (Fig.S2B), it is speculated ZNF507 dominantly functions in ectodermal regulation than mesodermal control. As DU145 is expresses the NE marker NeuroD1 and its transcription is regulated by the canonical TGF-β signaling mediator Smad3 [45, 46], it is plausible that ZNF507 functions in PC progression to the NE-like phenotype. Accumulating evidence suggests TGF-β signaling is involved in the development of PC or high-grade mCRPC through cancer cell cycle progression, EMT, and metastasis [29, 31, 47–49]. Our *in vivo* experimental studies, which displayed alteration of tumor growth and metastatic capacity affected by *ZNF507* knockdown, also support this hypothesis (Fig. 6, Fig.S8, Fig.S9, Fig.S11). As there was a limitation of *in vivo* metastasis models that injecting tumor cells intravenously does not perfectly recapitulate the metastatic cascade, they were sufficient to prove the physiological changes in cancer properties by *ZNF507* knockdown. Therefore, it is worth paying attention to ZNF507 as an attractive marker for aggressive high-grade PC.

The crosstalk between adenocarcinoma and NE-like PC and the underlying PC transformation to the NE-phenotype mechanism is complicated [50, 51]. Several studies have shown a link between NEPC and cell lineage plasticity, NE trans-differentiation, and regression to stemness by numerous factors [51–56]. Of these, the TGF-β signaling has been consistently linked with the NE-differentiation and EMT in NE-like cancers [57, 58]. It affects the NEPC progression, contributing neurogenesis, neural differentiation, and CNS development through diverse pathways [59–62]. Our current results indicate that ZNF507 may influence the extracellular matrix, embryonic differentiation, neurodevelopment, and immune response (Fig. 4). Reports about ZNF507 alteration during neurodevelopment also support our findings [16, 17]. Similar to ZNF507 [14, 15], TGF-β and its related immune activity has been linked to schizophrenia, suggesting an interaction in neurodevelopmental disorders [63, 64]. For these reasons, in addition to further studying ZNF507 gene in other cancers, studies on ZNF507 and TGF-β signaling in early and neurodevelopment are highly recommended. As such, several projects are currently underway that investigate the correlation in early development, neurogenesis, and the ZNF507.

## Conclusions

In conclusion, we demonstrated the pivotal ZNF507 effect in promoting PC progression to the aggressive state. We propose that ZNF507 contributes to the positive feedback loop in TGF-β signaling by regulating TGFBR1 and MAP3K8 activation for the progression of PC to the metastatic aggressive state, and this finding highlights its potential as a promising marker for the diagnosis of metastatic PC and high-grade mCRPC or NEPC (Fig. 6J).

## Supplementary Information

**Additional file 1: Supplemental Table 1. **The primers used for qRT-PCR and ChIP qRT-PCR analysis. **Supplemental Table 2. **The antibodies used for Western blot analysis and Immunofluorescence. **Supplemental Table 3. **Selected genes in the microarray data following the GO analysis. **Supplemental Figure 1. **Representative images of the expression of ZNF507 (red) and NeuroD1 (green) assessed by immunofluorescence in the hyperplasia and Gleason grade 4 PC tumor specimens (Scale bar = 100μm, blue: DAPI staining). Three independent experiments per each target were performed. **Supplemental Figure 2. **(A) Relative mRNA expression of ZNF507 in RWPE1, DU145, PC3, PC3M, and 22Rv1 cell lines measured by qRT-PCR. The data are presented as the Means ± SD from three independent experiments. *p < 0.05, **p < 0.01 versus RWPE1 cells as a control. (B) Representative images of protein expression data of ZNF507 in the RWPE1, DU145, PC3, PC3M, and 22Rv1 cell lines analyzed by western blot. β-actin was used as a normalization control. Three independent experiments per each target were performed. (C) Representative images of protein expression data from the nuclear and cytosol extraction sample of DU145 and 22Rv1 cells analyzed by western blot. LaminB1 was used as a normalization control for nuclear samples and β-tubulin was used as a normalization control for cytosol samples. Three independent experiments per each target were performed. (D) Representative images of fluorescent imaging stained with DAPI (blue), PI (red), and ZNF507 (green) (Scale bar = 50 µm). **Supplemental Figure 3. **(A) Representative protein expression data assessed by western blot for ZNF507 in scramble or shZNF507 DU145 or 22Rv1 cells, the lines selected from the qRT-PCR assessment of ZNF507 knockdown; shZNF507 #2 treated DU145 and 22Rv1 cells. (B) PCNA staining of the scramble or shZNF507 DU145 or 22Rv1 cells performed by immunocytochemistry (Scale bar = 50 µm). The graph below presents relative corrected total cell fluorescence (CTCF). At least 4 pictures from each sample were taken and the CTCF were calculated. (C) Colony formation of the shZNF507 treated DU145 or 22Rv1 cells performed by soft agar assay. The graph below presents colony number and relative diameter. Each experiment consists of four samples from the same group of cells were measured and the three independent experiments were conducted. (D) Representative images of the invasion and migration assay performed with the scramble or shZNF507 DU145 or 22Rv1 cells using trans-well plate (Scale bar = 50 µm). The graph below presents relative ratio of migration and invasion from the transwell migration and invasion assay. At least four pictures from each sample were taken and the three independent experiments were performed. (E) Representative images of protein expression data of E-cadherin, N-cadherin, Vimentin, Snail, and Slug in the scramble or shZNF507 DU145 or 22Rv1 cells assessed by western blot. β-actin was used as an endogenous control. (F) Cell cycle analysis was performed with scramble or shZNF507 DU145 or 22Rv1 cells by propidium iodide (PI) staining. The proportion of cells in each cycle was measured. The graph in the right panel indicates ratio of cell cycle population from cell cycle analysis. Three independent experiments were performed and at least four samples per group were measured in each experiment. (G) Representative images of the protein expression data of CyclinA1, CyclinB1, CyclinD1, CyclinE1, CDK2, CDK4, and CDK6 in the scramble or shZNF507 DU145 or 22Rv1 cells assessed by western blot. β-actin was used as an endogenous control. (H) Apoptosis analysis conducted by PI-Annexin V-FITC staining in the scramble or shZNF507 DU145 or 22Rv1 cells. The graph in the right panel indicates cell population ratio from the apoptosis analysis of the scramble or shZNF507 DU145 or 22Rv1 cells. Three independent experiments were performed and at least 4 samples per group were measured in each experiment. (I) Representative images of the protein expression data of Bax, Survivin, Bcl-xL, Bcl-2, Caspase3, cleaved-caspase3, PARP, and cleaved-PARP in the scramble or shZNF507 DU145 or 22Rv1 cells assessed by western blot. β-actin was used as an endogenous control. For all the data, at least three independent experiments were performed. *p< 0.05, **p < 0.01, ***p < 0.001 versus scramble control. **Supplemental Figure 4. **(A) Representative images of the protein expression data of TGFBR1, MAP3K8, and FURIN detected by western blot in the scramble and shZNF507 DU145 or 22Rv1 cells, the lines selected from the qRT-PCR assessment of ZNF507 knockdown; shZNF507 #2 treated DU145 and 22Rv1 cells. (B) Representative images of the protein expression data of canonical TGF-β signal proteins, Smad2, and Smad3 with their phosphorylated forms, assessed by western blot. β-actin was used as a normalization control. (C) Representative images of the protein expression data of non-canonical TGF-β signal proteins, RAS, MEK, phosphorylated-MEK, ERK, and phosphorylated-ERK, examined by western blot. β-actin was used as a normalization control. **Supplemental Figure 5. **(A), (B) Relative mRNA expression of TP53INP1, ANKRD1, SBDS, CNTN1, SCARB2 assessed by qRT-PCR analysis of the shZNF507 DU145 or 22Rv1 cells compared with their respective scramble cells. *GAPDH* was used as an endogenous normalization control. (C) Relative mRNA expression of TP53INP1, ANKRD1, SBDS, CNTN1, SCARB2 assessed by qRT-PCR analysis of the mock and ZNF507 overexpression (ZNF507 OE) DU145 cells compared with their respective scramble cells. *GAPDH* was used as an endogenous normalization control. The data are presented as the Means ± SD from three independent experiments. *p < 0.05 versus scramble or mock control. **Supplemental Figure 6. **(A) CCK-8 proliferation assay of the mock and ZNF507 overexpression (ZNF507 OE) DU145 cells. The OD value at 450 nm was determined. Each cell lines were prepared for three independent experiments with at least six samples per group. (B) Colony formation of the mock and ZNF507 OE DU145 cells performed by soft agar assay. The graph below indicates the number and diameter of the colonies. (C) Representative images of PCNA staining of the mock and ZNF507 OE cells performed by immunocytochemistry (Scale bar = 50 μm). The graph in the right panel presents relative corrected total cell fluorescence (CTCF). At least 4 pictures from each sample were taken and the CTCF were calculated. (D) Cell cycle analysis performed with mock or ZNF507 OE cells by propidium iodide (PI) staining. The proportion of cells in each cycle was calculated. The graph in the right panel presents the ratio of cell cycle population. Three independent experiments were performed and at least four samples per group were measured in each experiment. (E) Apoptosis analysis conducted by PI-Annexin V-FITC staining in the mock or ZNF507 OE DI145 cells. The graph in the right panel indicates cell population ratio from the apoptosis analysis. Three independent experiments were performed and at least 4 samples per group were measured in each experiment. (F) Representative images of the migration and invasion assay performed with the mock or ZNF507 OE DU145 cells using trans-well plate (Scale bar = 50 μm). The graphs below present relative ratio of migration and invasion of the data from the migration and invasion assay. At least 4 pictures from each sample were taken and the three independent experiments were performed. (G) Representative images of protein expression data of E-cadherin, N-cadherin, Vimentin, Snail, and Slug in the mock and ZNF507 OE DU145 cells. (H) Representative images of protein expression data of phosphorylated and total MEK and ERK in the mock and ZNF507 OE DU145 cells. (I) Representative images of protein expression data of phosphorylated and total Smad2 and Smad3 in the mock and ZNF507 OE DU145 cells. β-actin was used as a normalization control. Three independent experiments per each target were performed. **Supplemental Figure 7. **(A), (B) Primer sets were set following the ZNF507 ChIP-sequencing data (GSM3636297 and GSM3636298) from MCF-7 cell lines through the NCBI database. Relatively high fold-enrichment regions of the promoter area were selected for the ChIP-qPCR. **Supplemental Figure 8. **(A) Tumors extracted from the xenograft experiments performed with the scramble and shZNF507 DU145 cells injected into Balb/C nude mice (*n* = 6 for each group). Three tumor samples were used for the Western blot analysis, while other three samples were used for the IF. (B) Representative images of the immunostaining against Ki67 and cleaved-Caspase 3 in tumors, performed by immunofluorescence (Scale bar = 100 μm). (C) Representative images of the immunostaining against ZNF507, TGFBR1, MAP3K8, and FURIN in tumors, performed by immunofluorescence (Scale bar = 100 μm). (D) Representative western blot data of ZNF507, TGFBR1, MAP3K8, and FURIN expression in the xenografted tissues. β-actin served as a loading control. (E) Representative images of the expression of canonical TGF-β signal proteins, Smad2, Smad3, and their phosphorylated forms, assessed by western blot. β-actin was used as a normalization control. (F) Representative images of the expression data of non-canonical TGF-β signal proteins, RAS, MEK, and p-MEK, ERK, and p-ERK, examined by western blot. β-actin was used as a normalization control. For all the data, three independent experiments were conducted. **Supplemental Figure 9. **(A) Representative images of ZNF507 (red) and NeuroD1 (green) or Synaptophysin (red) and NeuroD1 (green) expression assessed by immunofluorescence in the scramble and shZNF507 xenografted tumors (Scale bar = 100μm, blue: DAPI staining). (B) Representative protein expression data of NeuroD1 and Synaptophysin assessed by western blot in the scramble and shZNF507 xenografted tumors. β-actin was used as a normalization control. For all the data, three independent experiments were conducted. **Supplemental Figure 10. **(A) Representative images of ZNF507 (red) and NeuroD1 (green) or Synaptophysin (red) and NeuroD1 (green) expression assessed by immunofluorescence in the mock and ZNF507 overexpression (ZNF OE) DU145 cells (Scale bar = 50μm, blue: DAPI staining). (B) Representative protein expression data of NeuroD1 and Synaptophysin assessed by western blot in the Mock and ZNF507 OE DU145 cells. β-actin was used as a normalization control. **Supplemental Figure 11. **(A) Tumors extracted from the xenograft experiments conducted with the mock or ZNF507 DU145 cells injected into BALB/C nude mice. Six mice were performed for experiment and three tumors were used for Western blot, while other three tumors were used for histological analysis. (B) Graph showing the average weight of the mock and ZNF507 OE DU145 xenografted tumors. (C) Representative western blot data of ZNF507, TGFBR1, MAP3K8, and FURIN expression in the mock and ZNF507 OE DU145 xenografted tumors. (D) Representative western blot data of RAS, p-MEK, MEK, p-ERK, and ERK expression in the mock and ZNF507 OE DU145 xenografted tumors. (E) Representative images of p-Smad2, Smad2, p-Smad3, and Smad3 in in the mock and ZNF507 OE DU145 xenografted tumor assessed by western blot. (F) Representative western blot data of NeuroD1 and Synaptophysin expression in the mock and ZNF507 OE DU145 xenografted tumors. For all the western blot, β-actin was used as a normalization control and three independent experiments were performed.

## Data Availability

All data supporting the conclusions of this article are included in the manuscript and supplementary information files.

## Abbreviations

ADT: Androgen deprivation therapy
mCRPC: Metastatic castration-resistant prostate cance
NEPC: Neuroendocrine prostate carcinoma
PA: Prostate adenocarcinoma
EMT: Epithelial-mesenchymal transition
GO: Gene ontology
GSEA: Gene set enrichment analysis
MSigDB: Molecular Signature Database
KEGG: Kyoto Encyclopedia of Genes and Genome
GENT: Gene Expression database of Normal and Tumor tissues
EMBL-EBI: The European Bioinformatics Institute
GEO: Gene Expression Omnibus
