# Supplementary material for: ZNF507 affects TGF-β signaling via TGFBR1 and MAP3K8 activation in the progression of prostate cancer to an aggressive state
Source: J Exp Clin Cancer Res. 2021 Sep 18;40:291. doi: 10.1186/s13046-021-02094-3 (PMC8449443; doi:10.1186/s13046-021-02094-3)
Supplement: Supplementary file 1 — Additional file 1: Supplemental Table 1. The primers used for qRT-PCR and ChIP qRT-PCR analysis. Supplemental Table 2. The antibodies used for Western blot analysis and Immunofluorescence. Supplemental Table 3. Selected genes in the microarray data following the GO analysis. Supplemental Figure 1. Representative images of the expression of ZNF507 (red) and NeuroD1 (green) assessed by immunofluorescence in the hyperplasia and Gleason grade 4 PC tumor specimens (Scale bar = 100μm, blue: DAPI staining). Three independent experiments per each target were performed. Supplemental Figure 2. (A) Relative mRNA expression of ZNF507 in RWPE1, DU145, PC3, PC3M, and 22Rv1 cell lines measured by qRT-PCR. The data are presented as the Means ± SD from three independent experiments. *p < 0.05, **p < 0.01 versus RWPE1 cells as a control. (B) Representative images of protein expression data of ZNF507 in the RWPE1, DU145, PC3, PC3M, and 22Rv1 cell lines analyzed by western blot. β-actin was used as a normalization control. Three independent experiments per each target were performed. (C) Representative images of protein expression data from the nuclear and cytosol extraction sample of DU145 and 22Rv1 cells analyzed by western blot. LaminB1 was used as a normalization control for nuclear samples and β-tubulin was used as a normalization control for cytosol samples. Three independent experiments per each target were performed. (D) Representative images of fluorescent imaging stained with DAPI (blue), PI (red), and ZNF507 (green) (Scale bar = 50 µm). Supplemental Figure 3. (A) Representative protein expression data assessed by western blot for ZNF507 in scramble or shZNF507 DU145 or 22Rv1 cells, the lines selected from the qRT-PCR assessment of ZNF507 knockdown; shZNF507 #2 treated DU145 and 22Rv1 cells. (B) PCNA staining of the scramble or shZNF507 DU145 or 22Rv1 cells performed by immunocytochemistry (Scale bar = 50 µm). The graph below presents relative corrected total cell fluorescenc [file 13046_2021_2094_MOESM1_ESM.docx]

**Supplemental Table. 1** The primers used for qRT-PCR and ChIP qRT-PCR analysis.

| **qRT-PCR** | | |
| --- | --- | --- |
| ZNF507 | F | 5’-gaattgtcagatgggcaggt-3’ |
|  | R | 5’-tcatcgtctgaagcgttttg-3’ |
| TGFBR1 | F | 5’-tcaggttctggctcaggttt-3’ |
|  | R | 5’-ttctccaaatcgacctttgc-3’ |
| MAP3K8 | F | 5’-ggccattcaaccaaagcaga-3’ |
|  | R | 5’-gataggctgagcgagggtag-3’ |
| FURIN | F | 5’-gactgggccttcatgacaac-3’ |
|  | R | 5’-ttggtcagcgtcccatagtt-3’ |
| Slug | F | 5’-cctttttcttgccctcactg-3’ |
|  | R | 5’-ttggagcagtttttgcactg-3’ |
| Twist1 | F | 5’-ttcctctaccaggtcctcca-3’ |
|  | R | 5’-atcctccagaccgagaagg-3’ |
| Snail | F | 5’-tttaccttccagcagcccta-3’ |
|  | R | 5’-ggacagagtcccagatgagc-3’ |
| KLF8 | F | 5’-ggtgtccacgtcaacatctg-3’ |
|  | R | 5’-atctgctggctaccagtgct-3’ |
| ZEB1 | F | 5’-aactgctgggaggatgacac-3’ |
|  | R | 5’-tctgcatctgactcgcattc-3’ |
| ZEB2 | F | 5’-gtaccttcagcgcagtgaca-3’ |
|  | R | 5’-caggtggcaggtcattttct-3’ |
| CDK1 | F | 5’-gaagcctagcatcccatgtc-3’ |
|  | R | 5’-ccattttgccagaaattcgt-3’ |
| CDK2 | F | 5’-gtggtgtggccaggagttac-3’ |
|  | R | 5’-cttcatccaggggaggtaca-3’ |
| CDK4 | F | 5’-ccgaagttcttctgcagtcc-3’ |
|  | R | 5’-ccacagaagagaggctttcg-3’ |
| CDK6 | F | 5’-accagcagcggacaaataaa-3’ |
|  | R | 5’-gctctgtaccacagcgtgac-3’ |
| CyclinA1 | F | 5’-tggagtctgaagcaatgcac-3’ |
|  | R | 5’-ggggctctggtgagtatctg-3’ |
| CyclinD1 | F | 5’-tggtgaacaagctcaagtgg-3’ |
|  | R | 5’-tgcggatgatctgtttgttc-3’ |
| CyclinE1 | F | 5’-gttgcaccagtttgcgtatg-3’ |
|  | R | 5’-aggggacttaaacgccactt-3’ |
| CDC25a | F | 5’-cccaaaggaaccattgagaa-3’ |
|  | R | 5’-cctgatgtttcccagcaact-3’ |
| TP53INP1 | F | 5'-agtcccagagtggaagctca-3' |
|  | R | 5'-agctcttgggttgttccaga-3' |
| ANKRD1 | F | 5'-gcttggaaggacatttggca-3' |
|  | R | 5'-agtggatggctgtggattca-3' |
| SBDS | F | 5'-aagttctgcagacccactca-3' |
|  | R | 5'-ggtcatctgttccaaacgca-3' |
| CNTN1 | F | 5'-cagattcggtattgggctgc-3' |
|  | R | 5'-tactgggtgtctggcagaag-3' |
| SCARB2 | F | 5'-gctgggtgtgttctttggtt-3' |
|  | R | 5'-agtcggggtagtggttgaag-3' |
| GAPDH | F | 5’-cagcctcaagatcatcagca-3’ |
|  | R | 5’-tgtggtcatgagtccttcca-3’ |
|  | | |
| **ChIP qRT-PCR** | | |
| TGFBR1 R1 | F | 5’-actggaactgagagggcaaa-3’ |
|  | R | 5’-caaactcccctcttgctcag-3’ |
| TGFBR1 R2 | F | 5’-agctgcggtgtagagcaagt-3’ |
|  | R | 5’-gaaaaggcgtggatattgga-3’ |
| TGFBR1 R3 | F | 5’-atcctggatctgtgctggag-3’ |
|  | R | 5’-ccaaacccagaaagtcctca-3’ |
| TGFBR1 R4 | F | 5’-cgcgcctagaggaggttag-3’ |
|  | R | 5’-tagagcgatgggtgtgtctg-3’ |
| MAP3K8 R1 | F | 5’-caatcatagctcacggcaga-3’ |
|  | R | 5’-gcgtgtcatcctagctcctc-3’ |
| MAP3K8 R2 | F | 5’-agaaactgcagagggagcac-3’ |
|  | R | 5’-ggcacaacccagattcaaac-3’ |
| MAP3K8 R3 | F | 5’-agggcgcgagtacactaaga-3’ |
|  | R | 5’-ggagttgcaagaggtggttg-3’ |

**Supplemental Table. 2** The antibodies used for Western blot analysis and Immunofluorescence.

| **Antibody** | | **Company** | | **Catalog number** |
| --- | --- | --- | --- | --- |
| **Primary** | ZNF507 (WB, 1:1,000; IF, 1:200)  (IF, 1:200) | Abcam  Novus Biologicals | ab85672  NBP1-82932 | |
|  | TGFBR1 (WB, 1:1,000; IF, 1:200) | Abcam | ab31013 | |
|  | MAP3K8 (WB, 1:1,000; IF, 1:200) | Abcam | ab137589 | |
|  | FURIN (WB, 1:1,000; IF, 1:200) | Invitrogen | PA1-062 | |
|  | NeuroD1 (WB, 1:1,000; IF, 1:200) | Abcam | ab60704 | |
|  | Synaptophysin (WB, 1:1,000; IF, 1:200) | Abcam | ab32127 | |
|  | PCNA (1:1,000) | Abcam | ab18197 | |
|  | Ki67 (1:250) | Abcam | ab15580 | |
|  | E-cadherin (1:1,000) | Cell Signaling Technology | #3195 | |
|  | N-cadherin (1:1,000) | Cell Signaling Technology | #13116 | |
|  | Vimentin (1:1,000) | Cell Signaling Technology | #5741 | |
|  | Snail (1:1,000) | Cell Signaling Technology | #3879 | |
|  | Slug (1:1,000) | Cell Signaling Technology | #9585S | |
|  | CyclinA1 (1:1,000) | Abcam | ab53699 | |
|  | CyclinB1 (1:1,000) | Cell Signaling Technology | #4135 | |
|  | CyclinD1 (1:1,000) | Cell Signaling Technology | #2922 | |
|  | CyclinE1 (1:1,000) | Cell Signaling Technology | #20808 | |
|  | CDK2 (1:1,000) | Cell Signaling Technology | #2546 | |
|  | CDK4 (1:1,000) | Cell Signaling Technology | #12790 | |
|  | CDK6 (1:1,000) | Cell Signaling Technology | #3136S | |
|  | Bax (1:1,000) | Cell Signaling Technology | #5023 | |
|  | Survivin (1:1,000) | Cell Signaling Technology | #2808 | |
|  | Bcl-xL (1:1,000) | Cell Signaling Technology | #2764 | |
|  | Bcl-2 (1:1,000) | Cell Signaling Technology | #3492 | |
|  | Caspase3 (1:1,000) | Cell Signaling Technology | #9662 | |
|  | cleaved Caspase3 (WB, 1:1,000; IF, 1:500) | Cell Signaling Technology | #9664 | |
|  | PARP (1:1,000) | Cell Signaling Technology | #9532 | |
|  | cleaved PARP (1:1,000) | Cell Signaling Technology | #5625 | |
|  | phospho-Smad2 (1:1,000) | Cell Signaling Technology | #18338 | |
|  | Smad2 (1:1,000) | Cell Signaling Technology | #5339 | |
|  | phospho-Smad3 (1:1,000) | Cell Signaling Technology | #9520 | |
|  | Smad3 (1:1,000) | Cell Signaling Technology | #9523 | |
|  | RAS (1:1,000) | Cell Signaling Technology | #3965 | |
|  | phospho-MEK (1:1,000) | Cell Signaling Technology | #9154 | |
|  | MEK (1:1,000) | Cell Signaling Technology | #9126 | |
|  | phospho-ERK (1:1,000) | Cell Signaling Technology | #4370 | |
|  | ERK (1:1,000) | Cell Signaling Technology | #4695 | |
|  | LaminB1 (1:1,000) | Invitrogen | PA5-19468 | |
|  | β-tubulin (1:1,000) | Cell Signaling Technology | #2128 | |
|  | β-actin (1:1,000) | Santa Cruz Biotechnology | sc-47778 | |
| **Secondary** | anti-mouse IgG (1:5,000) | Cell Signaling Technology | #7076S | |
|  | anti-rabbit IgG (1:5,000) | Cell Signaling Technology | #7074S | |

**Supplemental Table. 3** Selected genes in the microarray data following the GO analysis.

| **Gene symbol** | **Gene Description** | **Related GO annotation** |
| --- | --- | --- |
| TGFBR1 | Transforming growth factor, beta receptor 1 | Angiogenesis  Apoptotic process  Cell cycle  Cell death  Cell differentiation  Cell migration  Cell proliferation  Neurogenesis |
| MAP3K8 | Mitogen-activated kinase kinase kinase 8 | Cell Cycle |
| FURIN | Paired amino acid cleaving enzyme | Cell Death  Cell differentiation  Cell proliferation |
| TP53INP1 | Tumor protein p53 inducible nuclear protein 1 | Apoptotic process  Cell cycle  Cell death |
| ANKRD1 | Ankyrin repeat domain 1 | Cell differentiation  Neurogenesis |
| SBDS | Shwachman-Bodian-Diamond syndrome | Cell cycle  Cell migration  Cell proliferation  Neurogenesis |
| CNTN1 | Contactin 1 | Cell differentiation  Neurogenesis |
| SCARB2 | Scavenger receptor class B, member 2 | Cell differentiation |

**Supplemental Fig. S1**


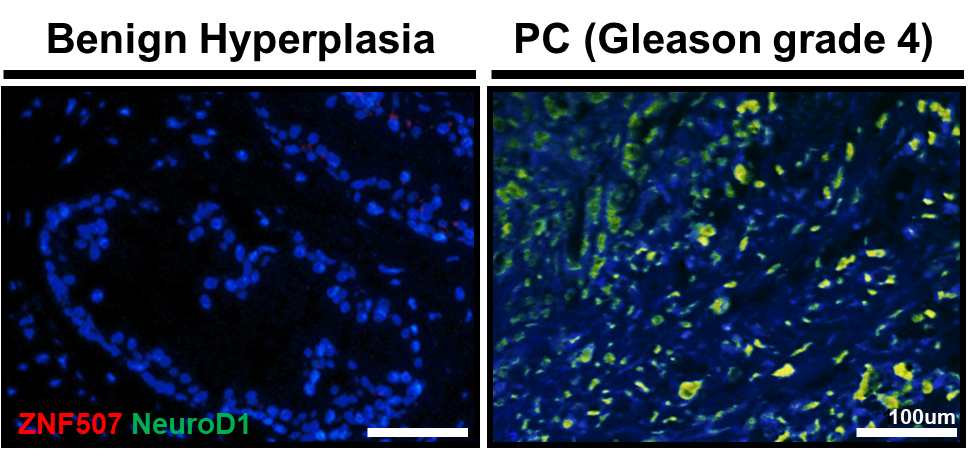


**Supplemental Figure. 1**

Representative images of the expression of ZNF507 (red) and NeuroD1 (green) assessed by immunofluorescence in the hyperplasia and Gleason grade 4 PC tumor specimens (Scale bar = 100μm, blue: DAPI staining). Three independent experiments per each target were performed.

**Supplemental Fig. S2**


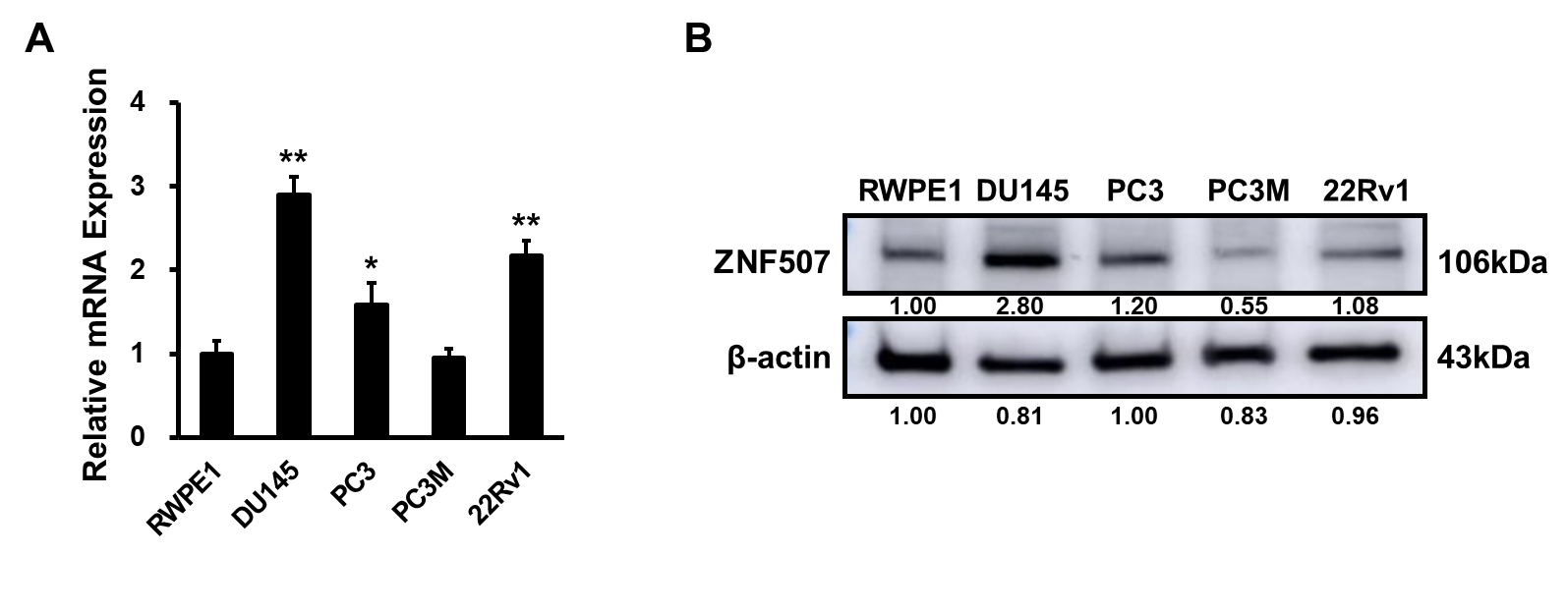


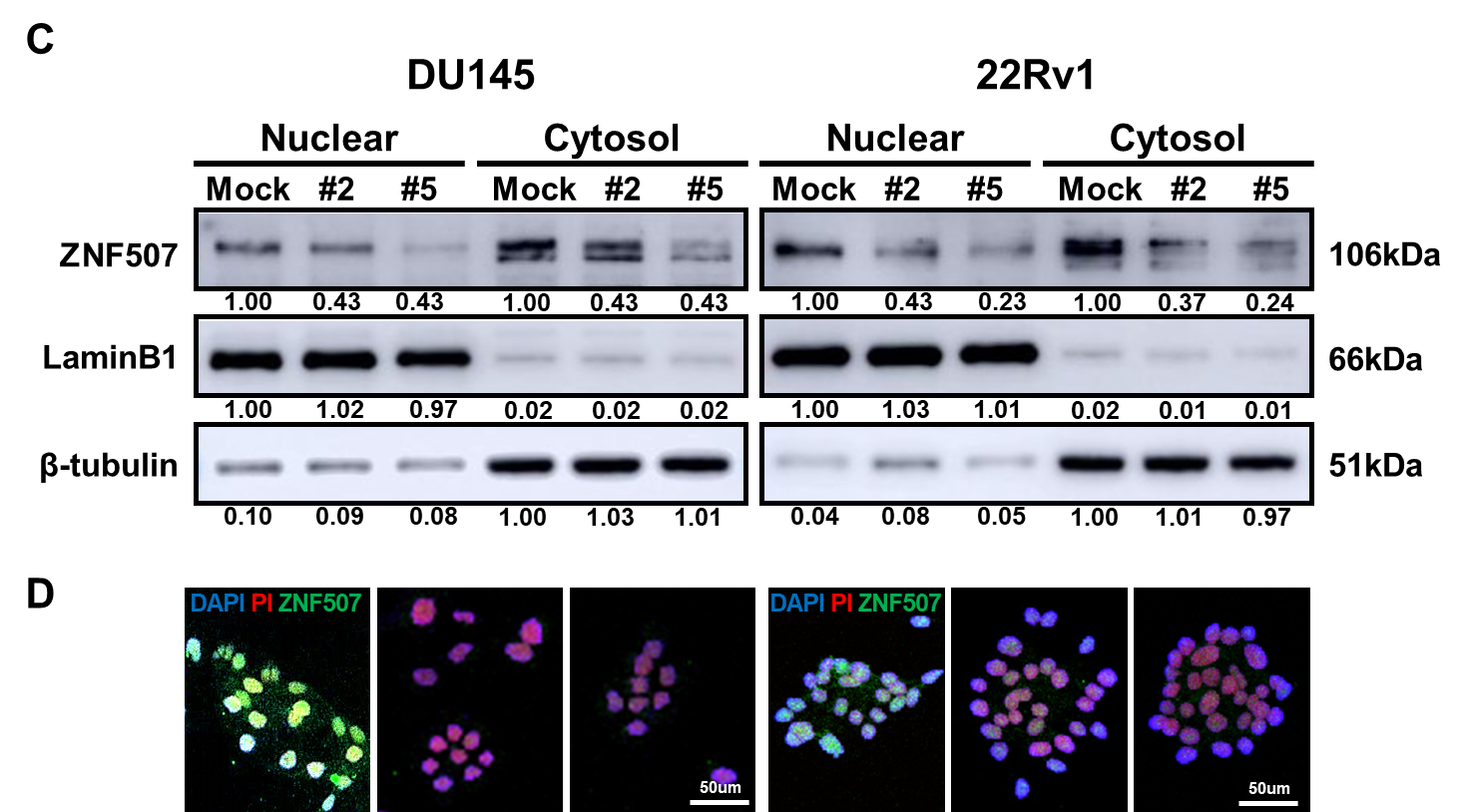


**Supplemental Figure. 2**

(A) Relative mRNA expression of ZNF507 in RWPE1, DU145, PC3, PC3M, and 22Rv1 cell lines measured by qRT-PCR. The data are presented as the Means ± SD from three independent experiments. *p < 0.05, **p < 0.01 versus RWPE1 cells as a control.

(B) Representative images of protein expression data of ZNF507 in the RWPE1, DU145, PC3, PC3M, and 22Rv1 cell lines analyzed by western blot. β-actin was used as a normalization control. Three independent experiments per each target were performed.

(C) Representative images of protein expression data from the nuclear and cytosol extraction sample of DU145 and 22Rv1 cells analyzed by western blot. LaminB1 was used as a normalization control for nuclear samples and β-tubulin was used as a normalization control for cytosol samples. Three independent experiments per each target were performed.

(D) Representative images of fluorescent imaging stained with DAPI (blue), PI (red), and ZNF507 (green) (Scale bar = 50 µm).

**Supplemental Fig. S3**


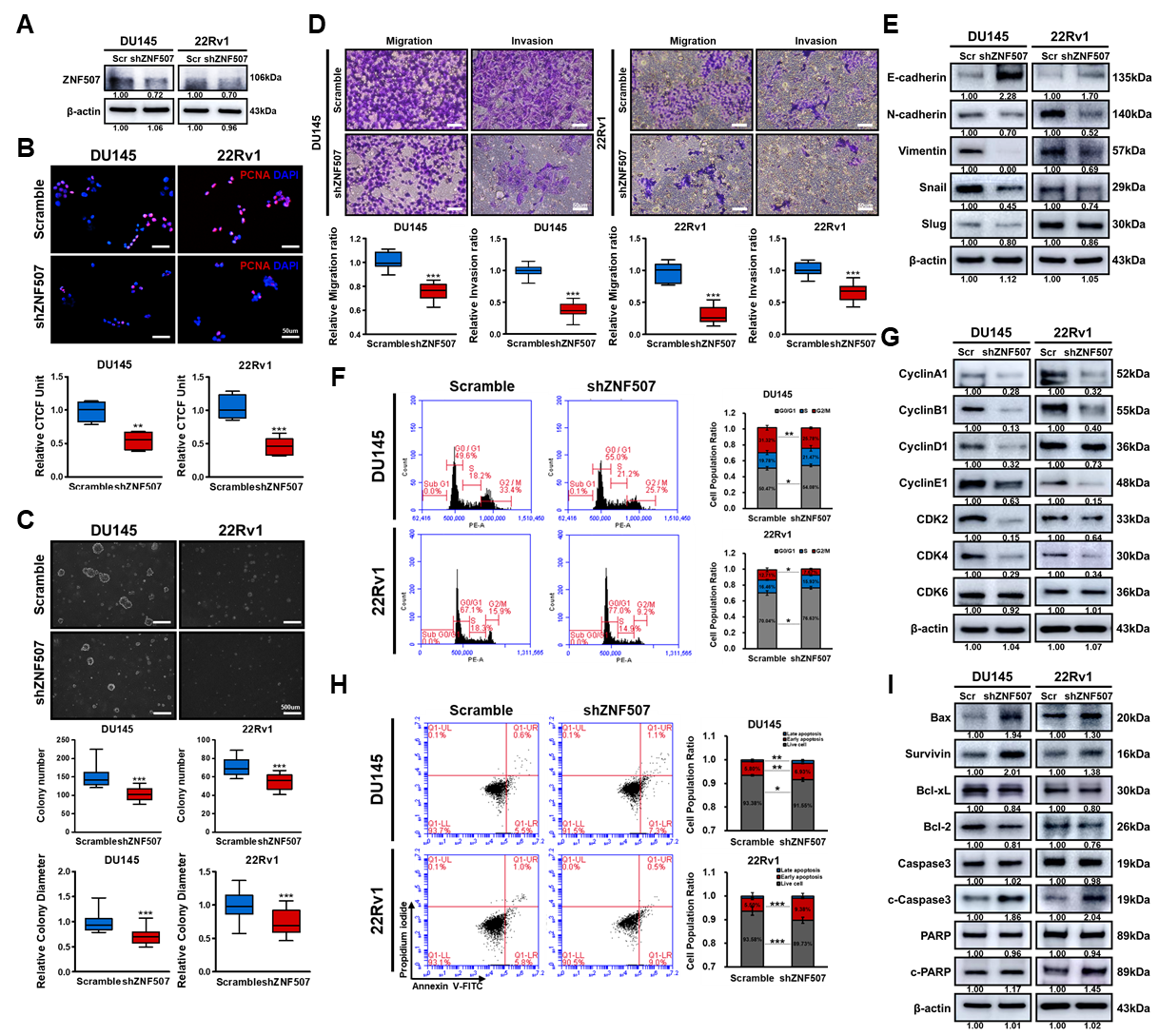


**Supplemental Figure. 3**

(A) Representative protein expression data assessed by western blot for ZNF507 in scramble or shZNF507 DU145 or 22Rv1 cells, the lines selected from the qRT-PCR assessment of ZNF507 knockdown; shZNF507 #2 treated DU145 and 22Rv1 cells.

(B) PCNA staining of the scramble or shZNF507 DU145 or 22Rv1 cells performed by immunocytochemistry (Scale bar = 50 µm). The graph below presents relative corrected total cell fluorescence (CTCF). At least 4 pictures from each sample were taken and the CTCF were calculated.

(C) Colony formation of the shZNF507 treated DU145 or 22Rv1 cells performed by soft agar assay. The graph below presents colony number and relative diameter. Each experiment consists of four samples from the same group of cells were measured and the three independent experiments were conducted.

(D) Representative images of the invasion and migration assay performed with the scramble or shZNF507 DU145 or 22Rv1 cells using trans-well plate (Scale bar = 50 µm). The graph below presents relative ratio of migration and invasion from the transwell migration and invasion assay. At least four pictures from each sample were taken and the three independent experiments were performed.

(E) Representative images of protein expression data of E-cadherin, N-cadherin, Vimentin, Snail, and Slug in the scramble or shZNF507 DU145 or 22Rv1 cells assessed by western blot. β-actin was used as an endogenous control.

(F) Cell cycle analysis was performed with scramble or shZNF507 DU145 or 22Rv1 cells by propidium iodide (PI) staining. The proportion of cells in each cycle was measured. The graph in the right panel indicates ratio of cell cycle population from cell cycle analysis. Three independent experiments were performed and at least four samples per group were measured in each experiment.

(G) Representative images of the protein expression data of CyclinA1, CyclinB1, CyclinD1, CyclinE1, CDK2, CDK4, and CDK6 in the scramble or shZNF507 DU145 or 22Rv1 cells assessed by western blot. β-actin was used as an endogenous control.

(H) Apoptosis analysis conducted by PI-Annexin V-FITC staining in the scramble or shZNF507 DU145 or 22Rv1 cells. The graph in the right panel indicates cell population ratio from the apoptosis analysis of the scramble or shZNF507 DU145 or 22Rv1 cells. Three independent experiments were performed and at least 4 samples per group were measured in each experiment.

(I) Representative images of the protein expression data of Bax, Survivin, Bcl-xL, Bcl-2, Caspase3, cleaved-caspase3, PARP, and cleaved-PARP in the scramble or shZNF507 DU145 or 22Rv1 cells assessed by western blot. β-actin was used as an endogenous control. For all the data, at least three independent experiments were performed. *p < 0.05, **p < 0.01, ***p < 0.001 versus scramble control.

**Supplemental Fig. S4**


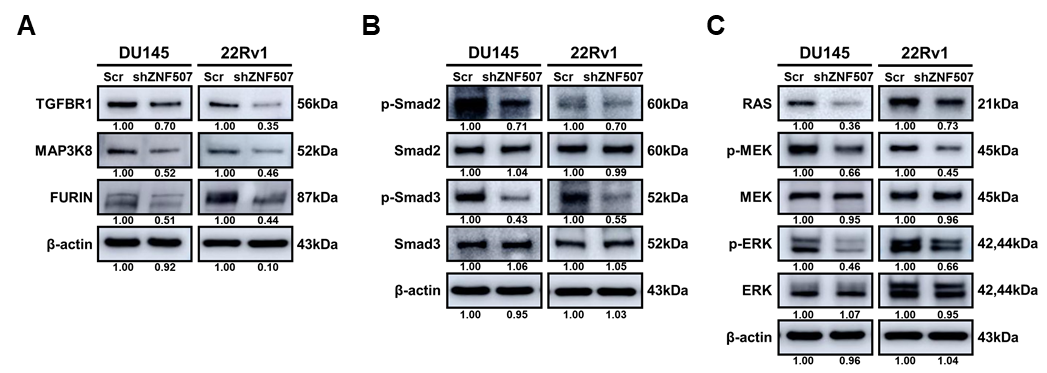


**Supplemental Figure. 4**

(A) Representative images of the protein expression data of TGFBR1, MAP3K8, and FURIN detected by western blot in the scramble and shZNF507 DU145 or 22Rv1 cells, the lines selected from the qRT-PCR assessment of ZNF507 knockdown; shZNF507 #2 treated DU145 and 22Rv1 cells.

(B) Representative images of the protein expression data of canonical TGF-β signal proteins, Smad2, and Smad3 with their phosphorylated forms, assessed by western blot. β-actin was used as a normalization control.

(C) Representative images of the protein expression data of non-canonical TGF-β signal proteins, RAS, MEK, phosphorylated-MEK, ERK, and phosphorylated-ERK, examined by western blot. β-actin was used as a normalization control.

**Supplemental Fig. S5**


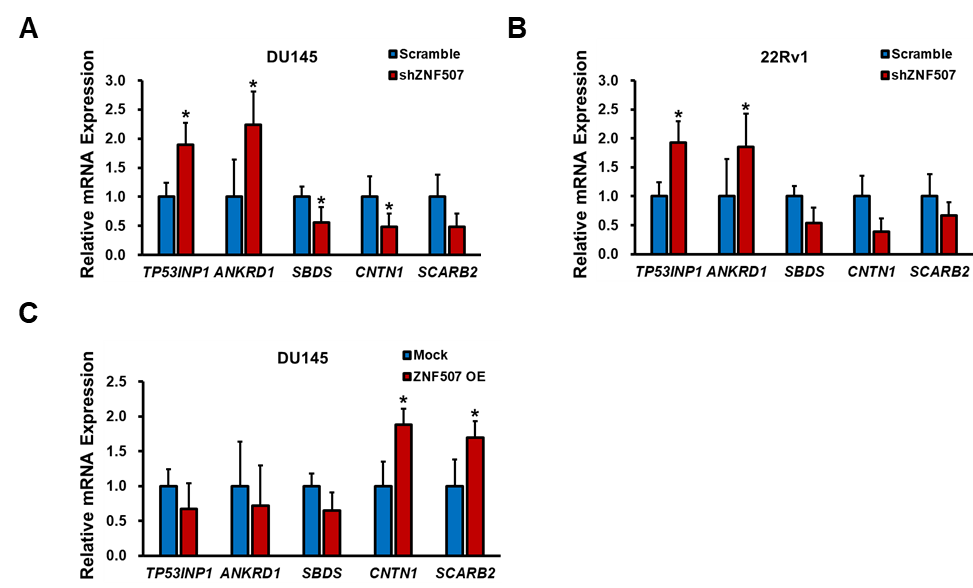


**Supplemental Figure. 5**

(A), (B) Relative mRNA expression of TP53INP1, ANKRD1, SBDS, CNTN1, SCARB2 assessed by qRT-PCR analysis of the shZNF507 DU145 or 22Rv1 cells compared with their respective scramble cells. *GAPDH* was used as an endogenous normalization control.

(C) Relative mRNA expression of TP53INP1, ANKRD1, SBDS, CNTN1, SCARB2 assessed by qRT-PCR analysis of the mock and ZNF507 overexpression (ZNF507 OE) DU145 cells compared with their respective scramble cells. *GAPDH* was used as an endogenous normalization control. The data are presented as the Means ± SD from three independent experiments. *p < 0.05 versus scramble or mock control.

**Supplemental Fig. S6**


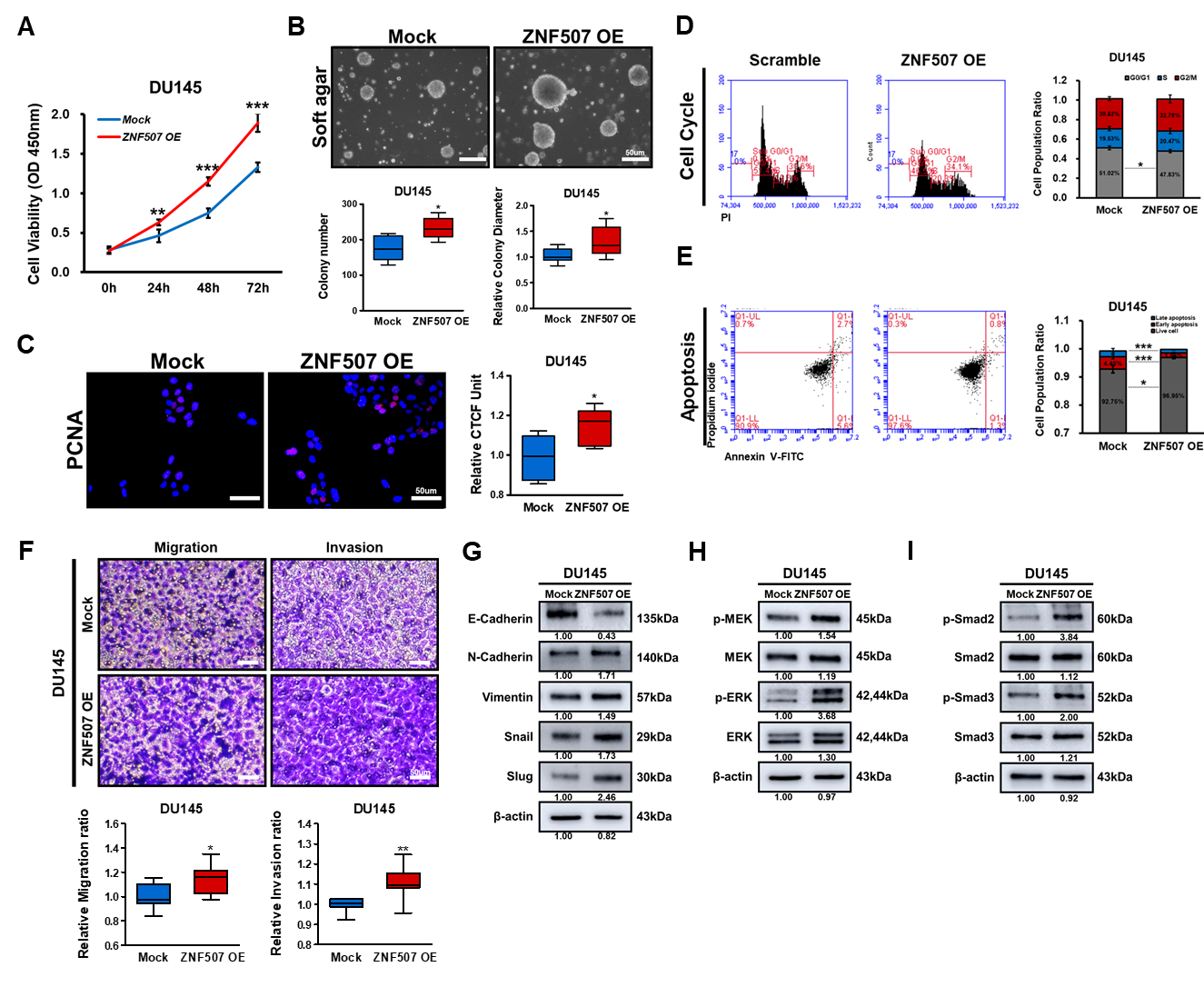


**Supplemental Figure. 6**

(A) CCK-8 proliferation assay of the mock and ZNF507 overexpression (ZNF507 OE) DU145 cells. The OD value at 450 nm was determined. Each cell lines were prepared for three independent experiments with at least six samples per group.

(B) Colony formation of the mock and ZNF507 OE DU145 cells performed by soft agar assay. The graph below indicates the number and diameter of the colonies.

(C) Representative images of PCNA staining of the mock and ZNF507 OE cells performed by immunocytochemistry (Scale bar = 50 μm). The graph in the right panel presents relative corrected total cell fluorescence (CTCF). At least 4 pictures from each sample were taken and the CTCF were calculated.

(D) Cell cycle analysis performed with mock or ZNF507 OE cells by propidium iodide (PI) staining. The proportion of cells in each cycle was calculated. The graph in the right panel presents the ratio of cell cycle population. Three independent experiments were performed and at least four samples per group were measured in each experiment.

(E) Apoptosis analysis conducted by PI-Annexin V-FITC staining in the mock or ZNF507 OE DI145 cells. The graph in the right panel indicates cell population ratio from the apoptosis analysis. Three independent experiments were performed and at least 4 samples per group were measured in each experiment.

(F) Representative images of the migration and invasion assay performed with the mock or ZNF507 OE DU145 cells using trans-well plate (Scale bar = 50 μm). The graphs below present relative ratio of migration and invasion of the data from the migration and invasion assay. At least 4 pictures from each sample were taken and the three independent experiments were performed.

(G) Representative images of protein expression data of E-cadherin, N-cadherin, Vimentin, Snail, and Slug in the mock and ZNF507 OE DU145 cells.

(B) Representative images of protein expression data of phosphorylated and total MEK and ERK in the mock and ZNF507 OE DU145 cells.

(C) Representative images of protein expression data of phosphorylated and total Smad2 and Smad3 in the mock and ZNF507 OE DU145 cells. β-actin was used as a normalization control. Three independent experiments per each target were performed.

**Supplemental Fig. S7**

**
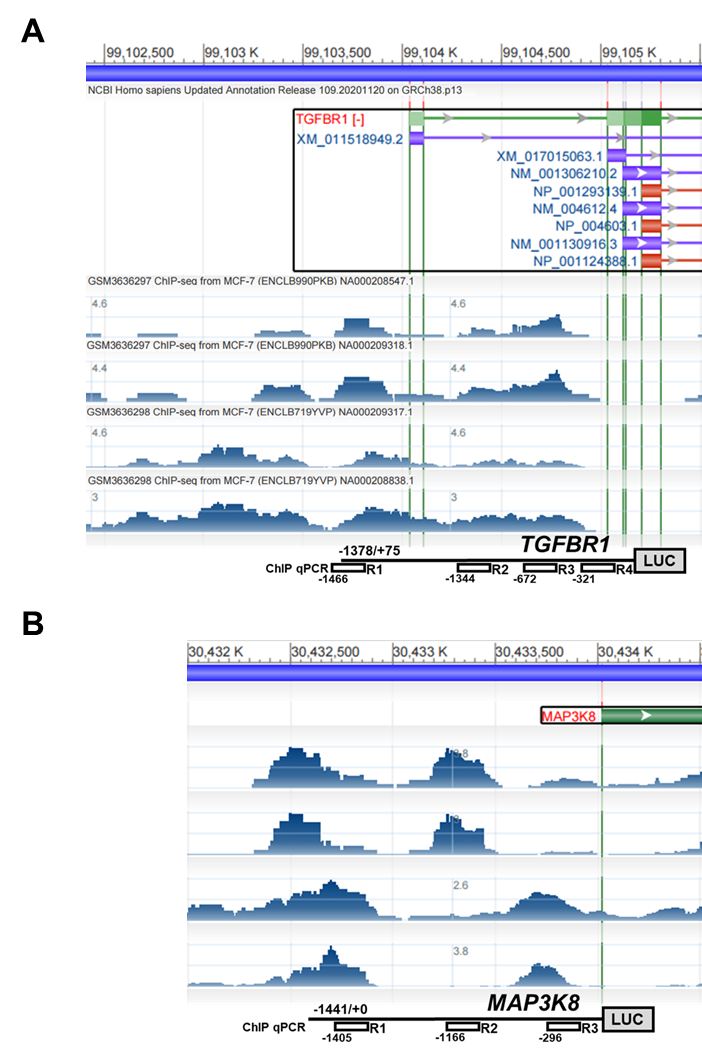
**

**Supplemental Figure. 7**

(A), (B) Primer sets were set following the ZNF507 ChIP-sequencing data (GSM3636297 and GSM3636298) from MCF-7 cell lines through the NCBI database. Relatively high fold-enrichment regions of the promoter area were selected for the ChIP-qPCR.

**Supplemental Fig. S8**


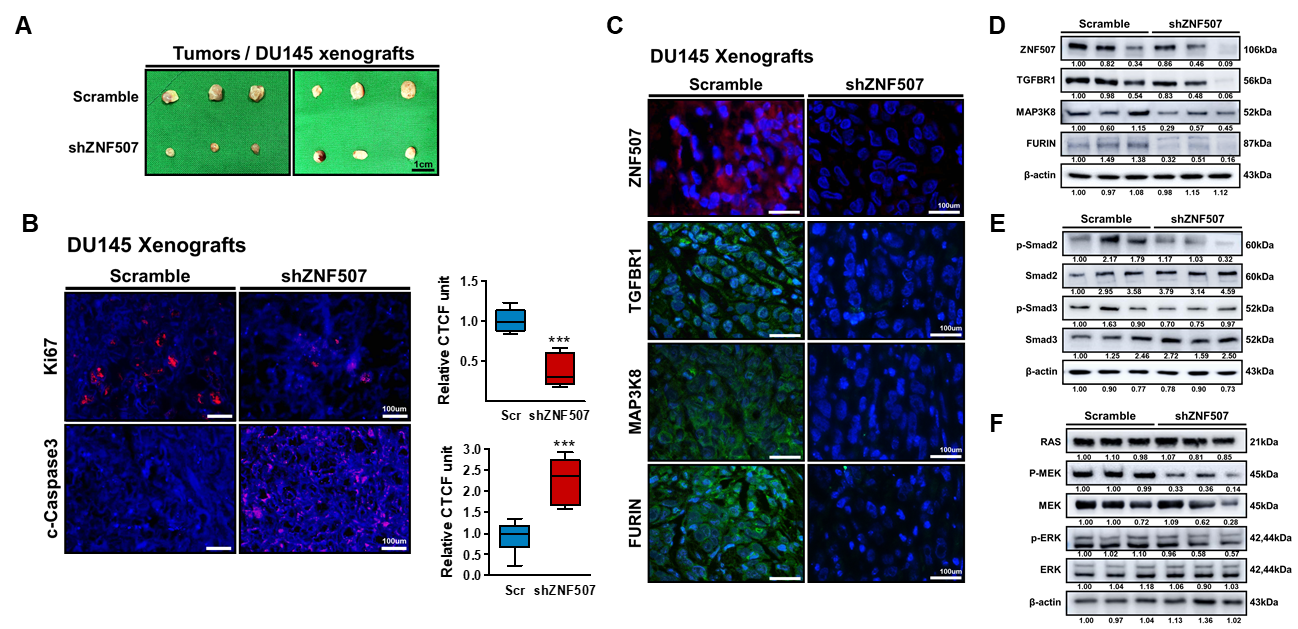


**Supplemental Figure. 8**

(A) Tumors extracted from the xenograft experiments performed with the scramble and shZNF507 DU145 cells injected into Balb/C nude mice (n = 6 for each group). Three tumor samples were used for the Western blot analysis, while other three samples were used for the IF.

(B) Representative images of the immunostaining against Ki67 and cleaved-Caspase 3 in tumors, performed by immunofluorescence (Scale bar = 100 μm).

(C) Representative images of the immunostaining against ZNF507, TGFBR1, MAP3K8, and FURIN in tumors, performed by immunofluorescence (Scale bar = 100 μm).

(D) Representative western blot data of ZNF507, TGFBR1, MAP3K8, and FURIN expression in the xenografted tissues. β-actin served as a loading control.

(E) Representative images of the expression of canonical TGF-β signal proteins, Smad2, Smad3, and their phosphorylated forms, assessed by western blot. β-actin was used as a normalization control.

(F) Representative images of the expression data of non-canonical TGF-β signal proteins, RAS, MEK, and p-MEK, ERK, and p-ERK, examined by western blot. β-actin was used as a normalization control. For all the data, three independent experiments were conducted.

**Supplemental Fig. S9**


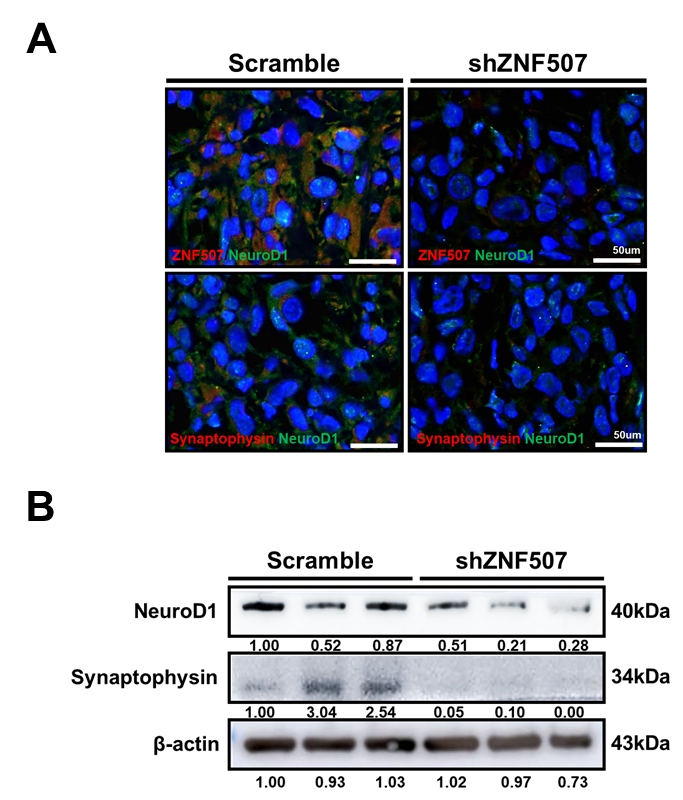


**Supplemental Figure. 9**

(A) Representative images of ZNF507 (red) and NeuroD1 (green) or Synaptophysin (red) and NeuroD1 (green) expression assessed by immunofluorescence in the scramble and shZNF507 xenografted tumors (Scale bar = 100μm, blue: DAPI staining).

(B) Representative protein expression data of NeuroD1 and Synaptophysin assessed by western blot in the scramble and shZNF507 xenografted tumors. β-actin was used as a normalization control. For all the data, three independent experiments were conducted.

**Supplemental Fig. S10**

**
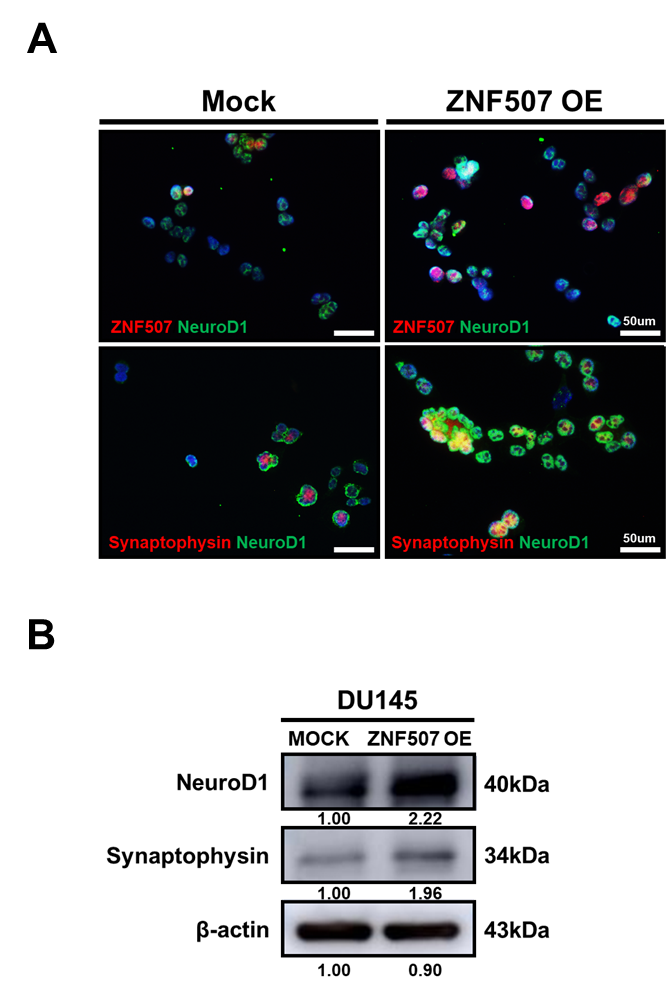
**

**Supplemental Figure. 10**

(A) Representative images of ZNF507 (red) and NeuroD1 (green) or Synaptophysin (red) and NeuroD1 (green) expression assessed by immunofluorescence in the mock and ZNF507 overexpression (ZNF OE) DU145 cells (Scale bar = 50μm, blue: DAPI staining).

(B) Representative protein expression data of NeuroD1 and Synaptophysin assessed by western blot in the Mock and ZNF507 OE DU145 cells. β-actin was used as a normalization control.

**Supplemental Fig. S11**


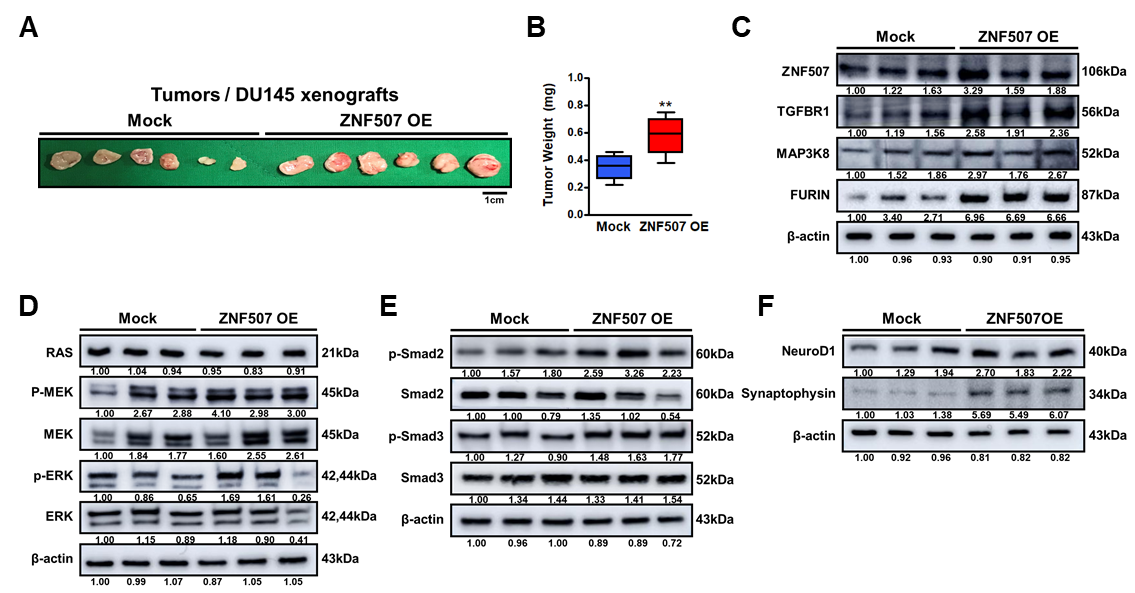


**Supplemental Figure. 11**

(A) Tumors extracted from the xenograft experiments conducted with the mock or ZNF507 DU145 cells injected into BALB/C nude mice. Six mice were performed for experiment and three tumors were used for Western blot, while other three tumors were used for histological analysis.

(B) Graph showing the average weight of the mock and ZNF507 OE DU145 xenografted tumors.

(C) Representative western blot data of ZNF507, TGFBR1, MAP3K8, and FURIN expression in the mock and ZNF507 OE DU145 xenografted tumors.

(D) Representative western blot data of RAS, p-MEK, MEK, p-ERK, and ERK expression in the mock and ZNF507 OE DU145 xenografted tumors.

(E) Representative images of p-Smad2, Smad2, p-Smad3, and Smad3 in in the mock and ZNF507 OE DU145 xenografted tumor assessed by western blot.

(F) Representative western blot data of NeuroD1 and Synaptophysin expression in the mock and ZNF507 OE DU145 xenografted tumors. For all the western blot, β-actin was used as a normalization control and three independent experiments were performed.
